# Porcine reproductive and respiratory syndrome virus infection triggers autophagy via ER stress-induced calcium signaling to facilitate virus replication

**DOI:** 10.1371/journal.ppat.1011295

**Published:** 2023-03-27

**Authors:** Feifei Diao, Chenlong Jiang, Yangyang Sun, Yanni Gao, Juan Bai, Hans Nauwynck, Xianwei Wang, Yuanqi Yang, Ping Jiang, Xing Liu

**Affiliations:** 1 Key Laboratory of Animal Disease Diagnostics and Immunology, Ministry of Agriculture, MOE International Joint Collaborative Research Laboratory for Animal Health & Food Safety, College of Veterinary Medicine, Nanjing Agricultural University, Nanjing, PR China; 2 Jiangsu Co-innovation Center for Prevention and Control of Important Animal Infectious Diseases and Zoonoses, Yangzhou University, Yangzhou, PR China; 3 Laboratory of Virology, Faculty of Veterinary Medicine, Ghent University, Merelbeke, Belgium; University of Iowa, UNITED STATES

## Abstract

Calcium (Ca^2+^), a ubiquitous second messenger, plays a crucial role in many cellular functions. Viruses often hijack Ca^2+^ signaling to facilitate viral processes such as entry, replication, assembly, and egress. Here, we report that infection by the swine arterivirus, porcine reproductive and respiratory syndrome virus (PRRSV), induces dysregulated Ca^2+^ homeostasis, subsequently activating calmodulin-dependent protein kinase-II (CaMKII) mediated autophagy, and thus fueling viral replication. Mechanically, PRRSV infection induces endoplasmic reticulum (ER) stress and forms a closed ER–plasma membrane (PM) contacts, resulting the opening of store operated calcium entry (SOCE) channel and causing the ER to take up extracellular Ca^2+^, which is then released into the cytoplasm by inositol trisphosphate receptor (IP3R) channel. Importantly, pharmacological inhibition of ER stress or CaMKII mediated autophagy blocks PRRSV replication. Notably, we show that PRRSV protein Nsp2 plays a dominant role in the PRRSV induced ER stress and autophagy, interacting with stromal interaction molecule 1 (STIM1) and the 78 kDa glucose-regulated protein 78 (GRP78). The interplay between PRRSV and cellular calcium signaling provides a novel potential approach to develop antivirals and therapeutics for the disease outbreaks.

## Author summary

Porcine reproductive and respiratory syndrome virus (PRRSV) infection results in a serious swine disease and causes a severe economic threat to the global swine industry. However, commercial vaccines do not provide satisfactory protection against PRRSV due to its genetic variation. Well understanding the mechanism of PRRSV replication is helpful to develop the effective strategies to control the disease. Calcium signaling has been reported to play an important role in the life cycles of viruses. In this study, we describe the picture that PRRSV infection induces ER stress, opens the store operated calcium entry (SOCE) channel and destroys host calcium homeostasis, further exploits CaMKII-AMPK-mTOR signaling mediated autophagy to benefit self-replication. Conversely, pharmacological inhibition ER stress or Ca^2+^ signaling suppresses PRRSV replication. Our work is the first report to link ER stress, Ca^2+^ signaling mediated autophagy, and PRRSV replication, which provides the new insights into virus-host interactions and controlling measures for future PRRSV outbreaks.

## Introduction

Porcine reproductive and respiratory syndrome virus (PRRSV) is a positive-sense single-stranded RNA virus belonging to the family *Arteriviridae* [1]. The genome is ~15 kb containing 11 open reading frames and encoding 14 nonstructural proteins and eight structural proteins [2,3]. Two polyproteins, PP1a and PP1ab, are encoded by ORF1a and ORF1b, respectively, and are processed into at least 14 nonstructural proteins (Nsps) by four ORF1a-encoded proteinases called Nsp1α, Nsp1β, Nsp2, and Nsp4, in which Nsp2 is the largest product [4–7]. Porcine reproductive and respiratory syndrome is an acute and highly contagious disease, causing large numbers of deaths in piglets and reproductive disorders in pregnant sows [8]. Currently, there are no fully effective vaccines or anti-viral drugs available for this disease [9]. To alleviate the burden of PRRS, greater research efforts in disease prevention and treatment that focus on the interactions between PRRSV and its host are urgently needed.

Calcium is a ubiquitous intracellular messenger responsible for myriad signal transduction processes, such as gene transcription, differentiation, proliferation and kinase activation [10]. Ca^2+^ and Ca^2+^ receptor proteins play essential roles in autophagy regulation, depending on cell type, cell status and Ca^2+^ signal [11]. Excessive release of intracellular Ca^2+^ from the ER activates calmodulin-dependent protein kinase-II (CaMKII) and the adenosine monophosphate-activated protein kinase (AMPK) signaling cascade, leading to mTOR signaling inhibition, and thus autophagy induction [12].

The ER is an important organelle for Ca^2+^ storage and protein processing and is highly sensitive to viral infection [13]. When the function of ER is disrupted, misfolded and unfolded proteins accumulate in the ER lumen and interfere with Ca^2+^ balance, resulting in ER stress [14], which in turn can induce autophagy [15–17]. A diverse array of viruses can induce autophagy [18], which can support or inhibit their replication depending on the virus and the host cell [19]. PRRSV infection induces autophagy, which promotes its replication [20,21]. In this study, we define the mechanism that PRRSV infection induces ER stress and disrupts of Ca^2+^ balance, finally activates autophagy through the CaMKII-AMPK-mTOR pathway to facilitate itself replication.

## Results

### PRRSV infection increases cytoplasmic Ca^2+^ levels

**Fig 1:**
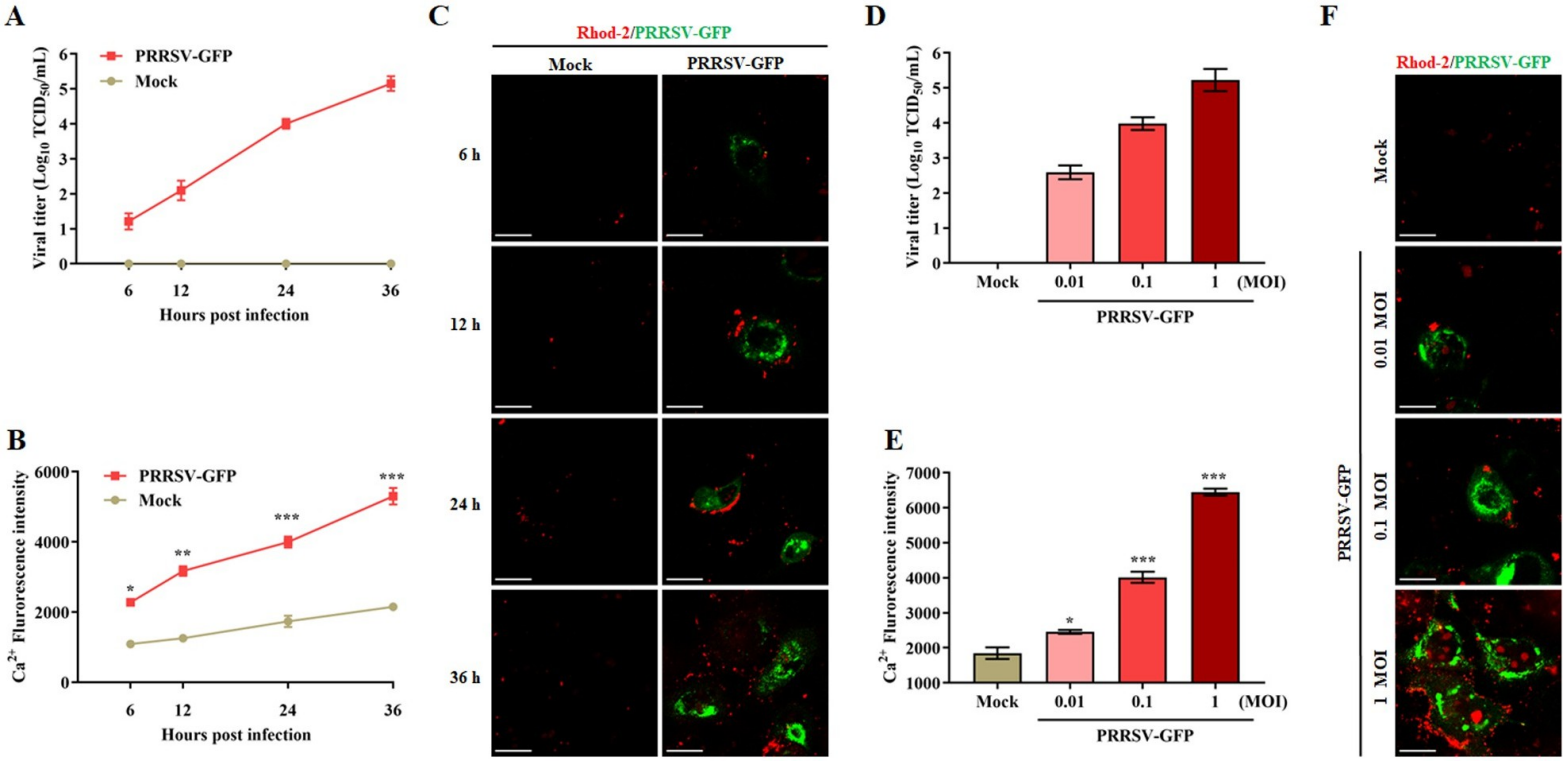
Infection with PRRSV results in increased cytoplasmic Ca^2+^. (A-C) Marc-145 cells were infected at MOI of 0.1 for different timepoints (6, 12, 24, 36 h). (**A**) PRRSV-GFP growth kinetics. (**B**) Increase in cytoplasmic Ca^2+^ over infection time course. (**C**) The fluorescence of Ca^2+^ (Rhod-2, Red) was observed using confocal microscopy after PRRSV-GFP infection at different timepoints. Scale bar, 10 μm. (D-F) Marc-145 cells were infected with PRRSV-GFP at different MOIs for 24 h. (**D**) Viral titers as a function of MOI at 24 hpi. (**E**) Cytoplasmic Ca^2+^ as a function of MOI. (**F**) The fluorescence of Ca^2+^ (Rhod-2, Red) was observed using confocal microscopy after PRRSV-GFP infection in different MOIs. Scale bar, 10 μm. Data are expressed as means ± SD (n = 3). *p<0.05; **p < 0.01; ***p < 0.001.

Several lines of evidence demonstrate that the host cell dysfunction caused by virus infection is accompanied by abnormal intracellular Ca^2+^ influx [22]. To determine whether PRRSV infection alters calcium homeostasis *in vitro*, we examined intracellular Ca^2+^ levels during PRRSV infection in Marc-145 cells. To visualize the correlation between PRRSV infection and changes in Ca^2+^ signaling, we conducted the experiments by using PRRSV-GFP. As shown in Fig 1A and 1B, in PRRSV-GFP infected cells, a progressive increase of Ca^2+^ in cytoplasm was detected from 6 h and peaked at 36 h, consistent with the kinetics of viral growth. Also, the intensity of Ca^2+^ fluorescence signaling, as visualized by Rhod-2 in red, was increased in PRRSV-GFP infected cells from 6 h to 36 h (Fig 1C). Not unexpectedly, intracellular Ca^2+^ levels in uninfected cells changed little over 36 h. Meanwhile, Ca^2+^ levels and Ca^2+^ fluorescence intensity in cytoplasm increased as a function of increased viral MOI (Fig 1D–1F). Consistently, the similar phenomena were also seen by using the wildtype PRRSV (S1 Fig). Taken together, these results suggest that PRRSV infection disrupts intracellular calcium homeostasis.

### Intracellular Ca^2+^ benefits PRRSV replication

**Fig 2:**
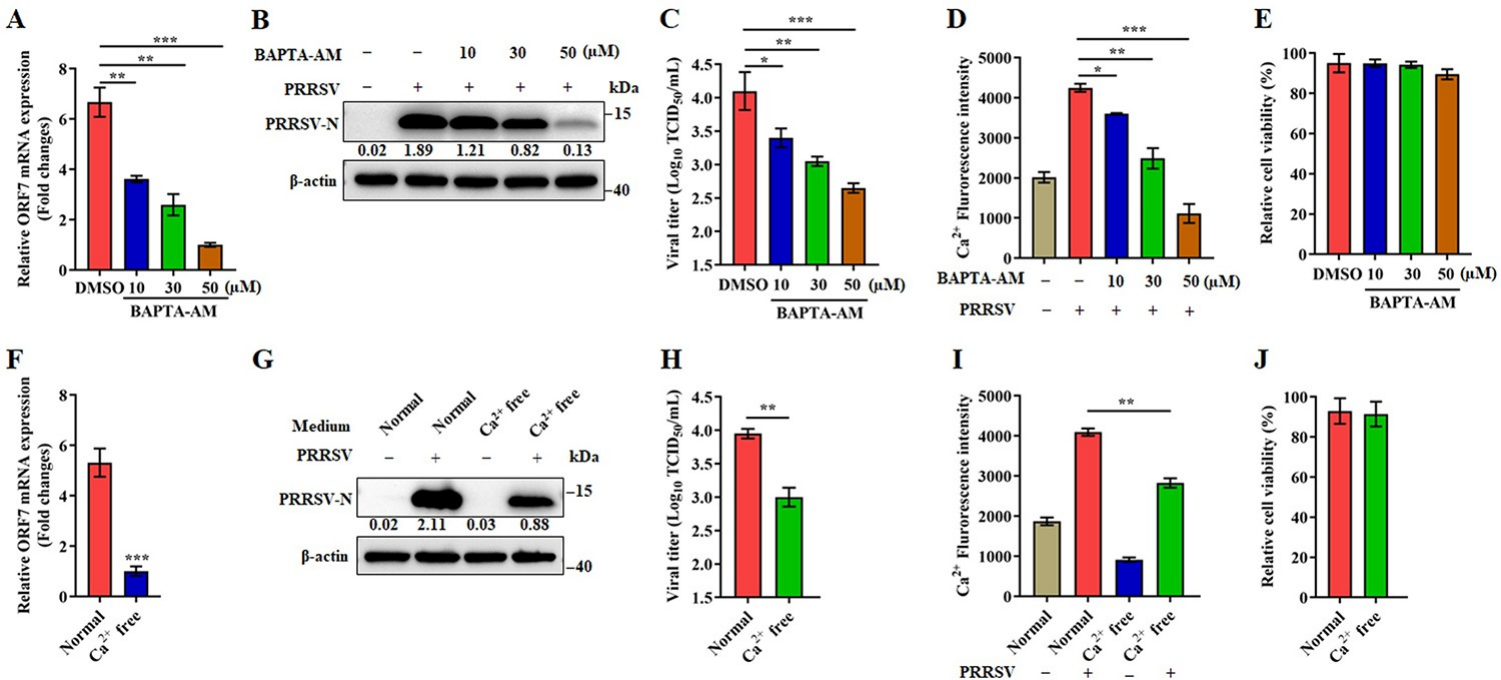
Intracellular Ca^2+^ benefits PRRSV replication. (A-E) BAPTA inhibits PRRSV infection. Marc-145 cells were mock or PRRSV infected (MOI = 0.1) for 24 h with different doses of BAPTA-AM. (**A**) RT-PCR for detection of PRRSV ORF7 (N protein). (**B**) Western blot of PRSSV N protein. (**C**) Viral titers as a function of BAPTA concentration. (**D**) Cytoplasmic Ca^2+^ induction after PRRSV infection, with and without BAPTA-AM. (**E**) Cell viability of Marc-145 cells treated with BAPTA-AM at indicated concentrations or DMSO for 24 h. (F-J) Exogenously supplied Ca^2+^ promotes PRRSV replication. Marc-145 cells were infected with PRRSV (MOI = 0.1) and then cultured in normal (Ca^2+^ = 1.80 mM) or Ca^2+^-free medium for 24 h. (**F**) RT-PCR for detection of PRRSV ORF7 (N protein). (**G**) Western blot of PRSSV N protein. (**H**) Viral titers as a function of exogenous Ca^2+^. (**I**) Cytoplasmic Ca^2+^ in cells after PRRSV infection in normal or Ca^2+^-free medium. (**J**) Cell viability of Marc-145 cells cultured with Ca^2+^-free or normal medium for 24 h. The protein levels were quantified by Image J and normalized to β-actin. Data are expressed as means ± SD. *p<0.05; **p < 0.01; ***p < 0.001. The experimental data are representative of results from three independent experiments.

The alteration of Ca^2+^ homeostasis in host cells is one of the strategies that some viruses use to favor their replication [23]. To determine the relationship between intracellular Ca^2+^ and PRRSV replication, we first evaluated replication in Marc-145 cells cultured in the presence of BAPTA-AM, a calcium chelator. As shown in Fig 2A and 2B, the amount of PRRSV-N (ORF7) mRNA and PRRSV-N protein decreased as the dose of BAPTA-AM increased as did the titer of PRRSV (Fig 2C). Fig 2D illustrates that cytoplasmic Ca^2+^ levels decreased as a function of BAPTA-AM dose in PRRSV infected cells. Cell viability was not affected at the dose of BAPTA-AM used in the experiment (Fig 2E). These data indicate that intracellular Ca^2+^ supports PRRSV replication. To further define the role of cytosolic Ca^2+^ in PRRSV replication, we observed PRRSV replication in Marc-145 cells cultured in calcium-normal or -free medium. The level of PRRSV-N (ORF7) mRNA and protein from infected cells cultured in calcium-free medium was significantly lower than that in the normal medium, as was the titer of PRRSV (Fig 2F–2H). In both type of media, PRRSV infected cells had significantly higher levels of intracellular Ca^2+^ than uninfected cells. However, the level of intracellular Ca^2+^ in the normal medium was significantly higher than that in calcium-free medium (Fig 2I). We found no difference in cell viability based on culture medium (Fig 2J). In addition, we determined the effects of BAPTA-AM or Ca^2+^ concentration on PRRSV entry. The results demonstrated that neither BAPTA-AM nor Ca^2+^ concentration had any effect on PRRSV adsorption or internalization (S2 Fig). Our results suggest that intracellular Ca^2+^ plays a positive role in PRRSV replication.

### Cytosolic Ca^2+^ promotes PRRSV replication through autophagy

Ca^2+^ signaling is critical for maintaining of cell homeostasis, but also for regulating the cell processes of autophagy and apoptosis [11]. Previous studies have shown that autophagy is activated during PRRSV infection and subsequently enhances viral replication in host cells [21]. Therefore, we hypothesized that intracellular Ca^2+^ benefits PRRSV replication via the autophagy pathway. To evaluate this, we first demonstrated that infection with live, but not UV-inactivated PRRSV, induced the formation of autophagosomes. The ratio of LC3II/I, a marker of autophagy, increased only with live virus infection as illustrated by western blotting (S3A Fig). And the autophagic plaques only detected in live virus infected cells (S3B and S3C Fig). Meanwhile, the ratio of LC3II/I and autophagic plaques increased as a function of increased viral MOI (S3D–S3F Fig). Next, we found that PRRSV replication increased in infected cells treated with rapamycin, an autophagy inducing drug, but decreased in cells treated with 3-MA, an autophagy inhibitor (S3G and S3H Fig). These results suggest that autophagy plays a positive role in PRRSV replication.

**Fig 3:**
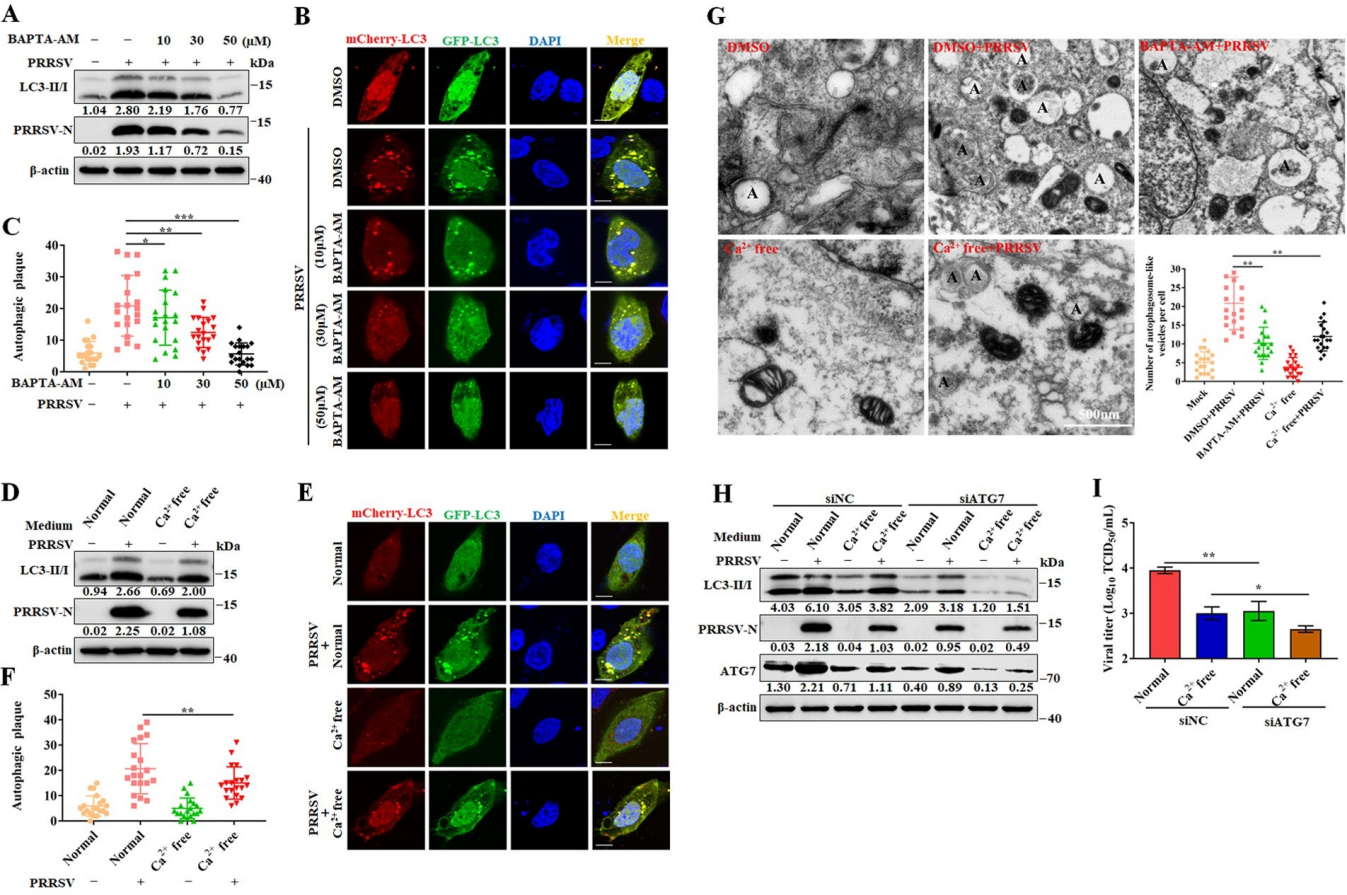
Cytosolic Ca^2+^ promotes PRRSV replication through autophagy. (A-C) BAPTA-AM inhibits PRRSV induced autophagy. Marc-145 cells were infected with PRRSV (MOI = 0.1) and treated with BAPTA-AM for 24 h. (**A**) The ratio of endogenous LC3I to LC3II determined by western blotting. (**B**) Marc-145 cells were transfected with p-mCherry-GFP-LC3 for 24 h and then infected with PRRSV (MOI = 0.1) and treated with BAPTA-AM for another 24 h. LC3 puncta were visualized by confocal microscopy. Nuclei were stained with DAPI (blue). Scale bar = 5 μm. (**C**) Quantitation of LC3 puncta formation. Results represent the number of LC3 puncta per cell in panel B (n  =  20). (D-F) Extracellular Ca^2+^ promotes PRRSV induced autophagy. (**D**) Marc-145 cells were infected with PRRSV (MOI = 0.1) and cultured in normal or Ca^2+^-free medium for 24 h. The ratio of endogenous LC3I to LC3II determined by western blotting. (**E**) LC3 puncta were visualized by confocal microscopy. Scale bar, 5 μm. (**F**) Quantitation of LC3 puncta formation. Results represent the number of LC3 puncta per cell in panel E (n  =  20). (**G**) The transmission electron microscopy analysis of virus-infected cells. Marc-145 cells were treated as shown in different groups (DMSO, DMSO + PRRSV, BAPTA-AM + PRRSV, Ca^2+^-free medium, Ca^2+^-free medium + PRRSV). 24 h later, cells were fixed and processed for electron microscopy analysis. A indicated the single- and double-membrane vesicles of autophagosome. The numbers of autophagosome were quantified from 20 different images in each group. (H-I) Marc-145 cells were transfected with control siRNA (siNC) or siRNA targeting ATG7 (siATG7) for 24 h, and then infected with PRRSV (MOI = 0.1) for another 24 h in normal or Ca^2+^-free medium. (**H**) PRRSV-N protein levels and the LC3-I/II ratio was determined by western blotting. (**I**) TCID_50_ of PRRSV in cell supernatants. The protein levels were quantified by Image J and normalized to β-actin. Data are expressed as means ± SD, n = 3 in G or n = 20 in C, F and G. *p<0.05; **p < 0.01; ***p < 0.001. The data are representative of results from three independent experiments.

Next, we detected the autophagy formation in the presence of BAPTA-AM after PRRSV infection. We showed that PRRSV induced autophagy was inhibited, in a dose dependent manner, in infected cells treated with BAPTA-AM, a calcium chelator (Fig 3A). By confocal microscopy we showed that autophagic plaque formation also decreased in a BAPTA-AM dose dependent manner (Fig 3B and 3C). Consistently, the level of LC3II and number of autophagic plaques were significantly lower in cells cultured in calcium free medium than those in normal cells after PRRSV infection (Fig 3D, 3E and 3F). Meanwhile, the transmission electron microscopy (TEM) was performed to detect the PRRSV-induced autophagosome formation with BAPTA-AM treatment or calcium free medium culture. The autophagosomes were obviously decreased in PRRSV infection treated with BAPTA-AM (BAPTA-AM + PRRSV) or calcium free medium (Ca^2+^ free + PRRSV) compared to those in the PRRSV infection with DMSO treatment (DMSO + PRRSV) (Fig 3G). These results support the relationship between PRRSV induced autophagy and intracellular Ca^2+^ levels.

Autophagy-related gene 7 (*ATG7*) is a crucial adaptor in the autophagy process [24]. To further explore the relationship between PRRSV induced autophagy and intracellular Ca^2+^, we examined the autophagy process induced by PRRSV infection by knocking down ATG7 in the cells cultured in normal or calcium free medium. The expression of LC3II/I decreased in ATG7 knockdown cells (Figs 3H and S4), indicating inhibition of the autophagy process. Importantly, the difference in PRRSV replication between the cells with and without calcium ion was insignificant (Fig 3H and 3I). These results indicate the intracellular Ca^2+^ facilitates PRRSV replication through the autophagy pathway.

### PRRSV induces autophagy through the CaMKII-AMPK-mTOR pathway

**Fig 4:**
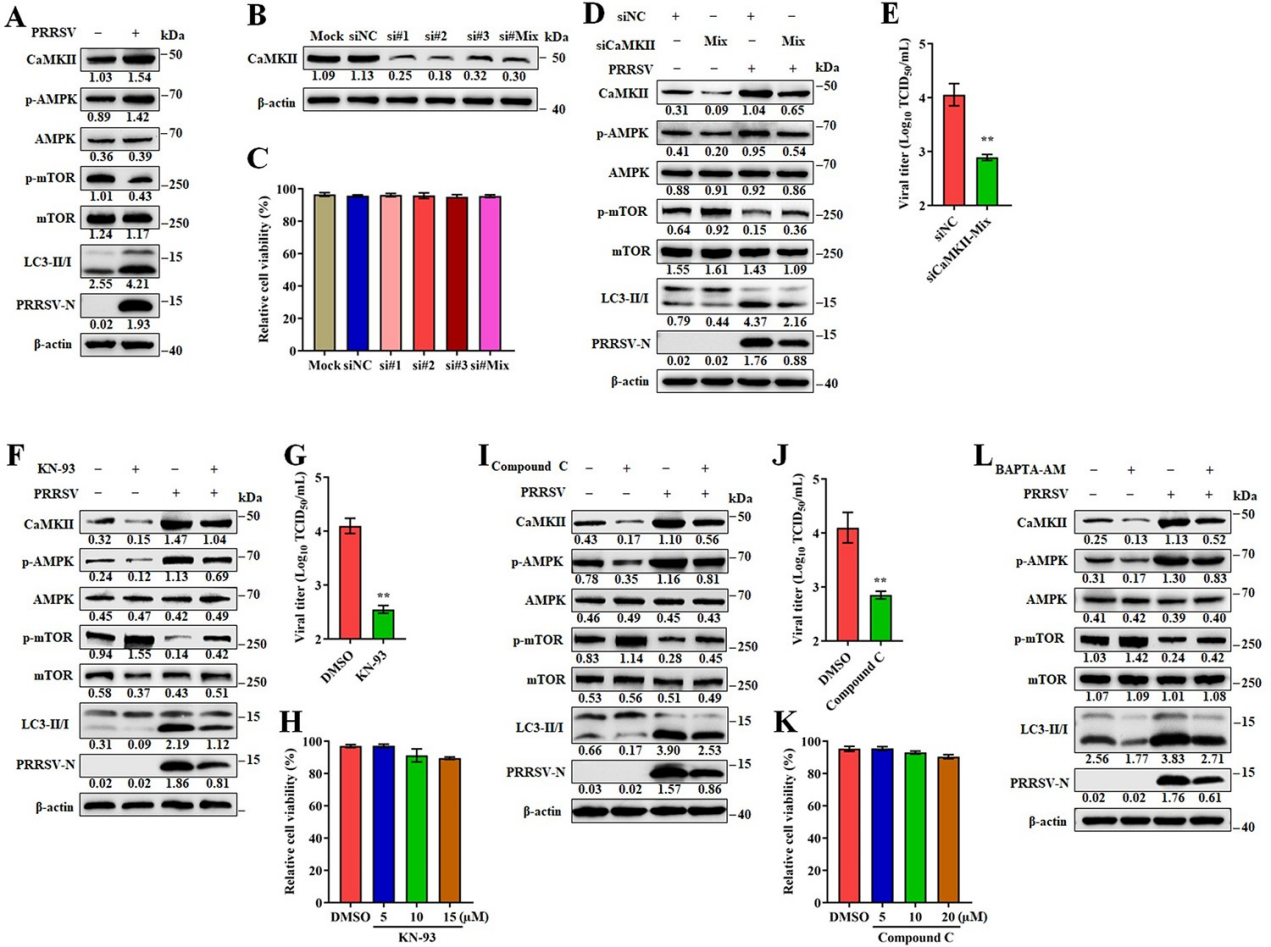
PRRSV induces autophagy through CaMKII-AMPK-mTOR pathway. (**A**) Marc-145 cells were mock or infected with PRRSV (MOI = 0.1) for 24 h. Cell lysates were analyzed by western blotting for CaMKII, p-AMPK, AMPK, p-mTOR, mTOR, LC3-I/II, PRRSV-N, and β-actin. (B-E) CaMKII mediated autophagy is essential for PRRSV efficient replication. (**B**) Western blotting was used to quantitate the level of CaMKII in siRNA1-, siRNA2-, siRNA3-, Mix (the equal amounts of siRNA1, siRNA2 and siRNA3) or siNC-transfected Marc-145 cells. (**C**) The cell viability of Marc-145 cells transfected with in siRNA1, siRNA2, siRNA3 or Mix (the equal amounts of siRNA1, siRNA2 and siRNA3) targeting to CaMKII, or siNC. (D-E) Marc-145 cells were transfected with siRNA Mix (the equal amounts of siRNA1, siRNA2 and siRNA3) targeting to CaMKII or siNC for 24 h, and mock or infected with PRRRV (MOI = 0.1) for another 24 h. (**D**) Cell lysates were prepared and analyzed by immunoblotting using anti-CaMKII, anti-p-AMPK, anti-AMPK, anti-p-mTOR, anti-mTOR, anti-LC3-I/II, anti PRRSV-N, and anti-β-actin antibodies. (**E**) TCID_50_ of PRRSV in cell supernatants. (F-H) Marc-145 cells were infected with PRRSV (MOI = 0.1) and treated with KN-93 (10 μM) or DMSO treatment for 24 h. (**F**) Cell lysates were analyzed by western blotting for CaMKII, p-AMPK, AMPK, p-mTOR, mTOR, LC3-I/II, PRRSV-N, and β-actin. (**G**) TCID_50_ of PRRSV in cell supernatants. (**H**) The effect of KN-93 on Marc-145 cell viability. Marc-145 cells were treated with KN-93 at indicated concentrations for 24 h, and then analyzed with CCK-8 system. (I-K) Marc-145 cells were infected with PRRSV (MOI = 0.1) and treated with Compound C (10 μM) or DMSO for 24 h. (**I**) Cell lysates were analyzed by western blotting for CaMKII, p-AMPK, AMPK, p-mTOR, mTOR, LC3-I/II, PRRSV-N, and β-actin. (**J**) TCID_50_ of PRRSV in cell supernatants. (**K**) The effect of Compound C on Marc-145 cell viability. Marc-145 cells were treated with Compound C at indicated concentrations for 24 h, and then analyzed with CCK-8 system. (**L**) Marc-145 cells were infected with PRRSV (MOI = 0.1) with or without BAPTA-AM (30 μM) for 24 h. Cell lysates were analyzed by western blotting for CaMKII, p-AMPK, AMPK, p-mTOR, mTOR, LC3-I/II, PRRSV-N, and β-actin. The protein levels were quantified by Image J and normalized to β-actin. The data are representative of results from three independent experiments. Error bars indicate the mean (± SD) of three independent experiments. *, p < 0.05; **, p < 0.01; and ***, p < 0.001.

Diverse Ca^2+^ mobilizing agents increase intracellular Ca^2+^ levels and activate pro-survival autophagy by activating Ca^2+^/calmodulin-dependent protein kinase 2 (CaMKII) [11]. An increase in cytoplasmic Ca^2+^ promotes activation of CaMKII, which, in turn, activates autophagy through the regulation of the AMPK/mTOR pathway [12]. We reasoned that PRRSV infection induces intracellular Ca^2+^ flux and activates autophagy through the CaMKII-AMPK-mTOR pathway. As shown in Figs 4A and S5A, PRRSV infection resulted in increased expression of CaMKII, phosphorylated AMPK (p-AMPK), and LC3II, and decreased expression of phosphorylated mTOR (p-mTOR). These results demonstrate that the CaMKII-AMPK-mTOR pathway is activated by PRRSV infection.

To further define the significance of CaMKII-AMPK-mTOR pathway in PRRSV replication, we treated the cells with inhibitors of CaMKII or AMPK then monitored the PRRSV induced autophagy and replication. To determine the effect of CaMKII on PRRSV replication, we designed small-interfering RNAs that targeted CaMKII. The results showed that all three CaMKII siRNA were efficient (Fig 4B). Meanwhile, these small-interfering RNAs have no effects on the cell viability (Fig 4C). Further, using siCaMKII-Mix (equal amounts of each three siRNA targeting CaMKII) to reduce CaMKII expression, we found that CaMKII expression played a positive role in PRRSV induced autophagy and replication (Figs 4D, 4E and S5B). The similar results were also observed when using a single siCaMKII (S6 Fig). Subsequently, we determined the effect of the specific CaMKII inhibitor, KN-93 on PRRSV replication. As shown in Figs 4F and S5C, autophagy induced by PRRSV was weakened in cells treated with KN-93, a specific CaMKII inhibitor, and as expected PRRSV replication was inhibited (Fig 4G). Similar results were seen with infected cells treated with Compound C, an AMPK inhibitor (Figs 4I, 4J and S5D). KN-93 and Compound C showed no cytotoxicity at the concentrations used in the experiments (Fig 4H and 4K). Consistent with these results, PRRSV induced autophagy and replication were both decreased in cells treated with BAPTA-AM, which has been shown to downregulate intracellular Ca^2+^(Figs 4L and S5E). Importantly, in BAPTA-AM treated infected cells, the CaMKII-AMPK-mTOR pathway was inhibited, with decreased levels of CaMKII, phosphorylated AMPK and increased levels phosphorylated mTOR. Altogether, these data demonstrate the increased intracellular Ca^2+^ during PRRSV infection activates autophagy through the CaMKII-AMPK-mTOR pathway, which in turn promotes PRRSV replication.

### PRRSV infection induces ER stress and utilizes SOCE channel to uptake extracellular calcium

**Fig 5:**
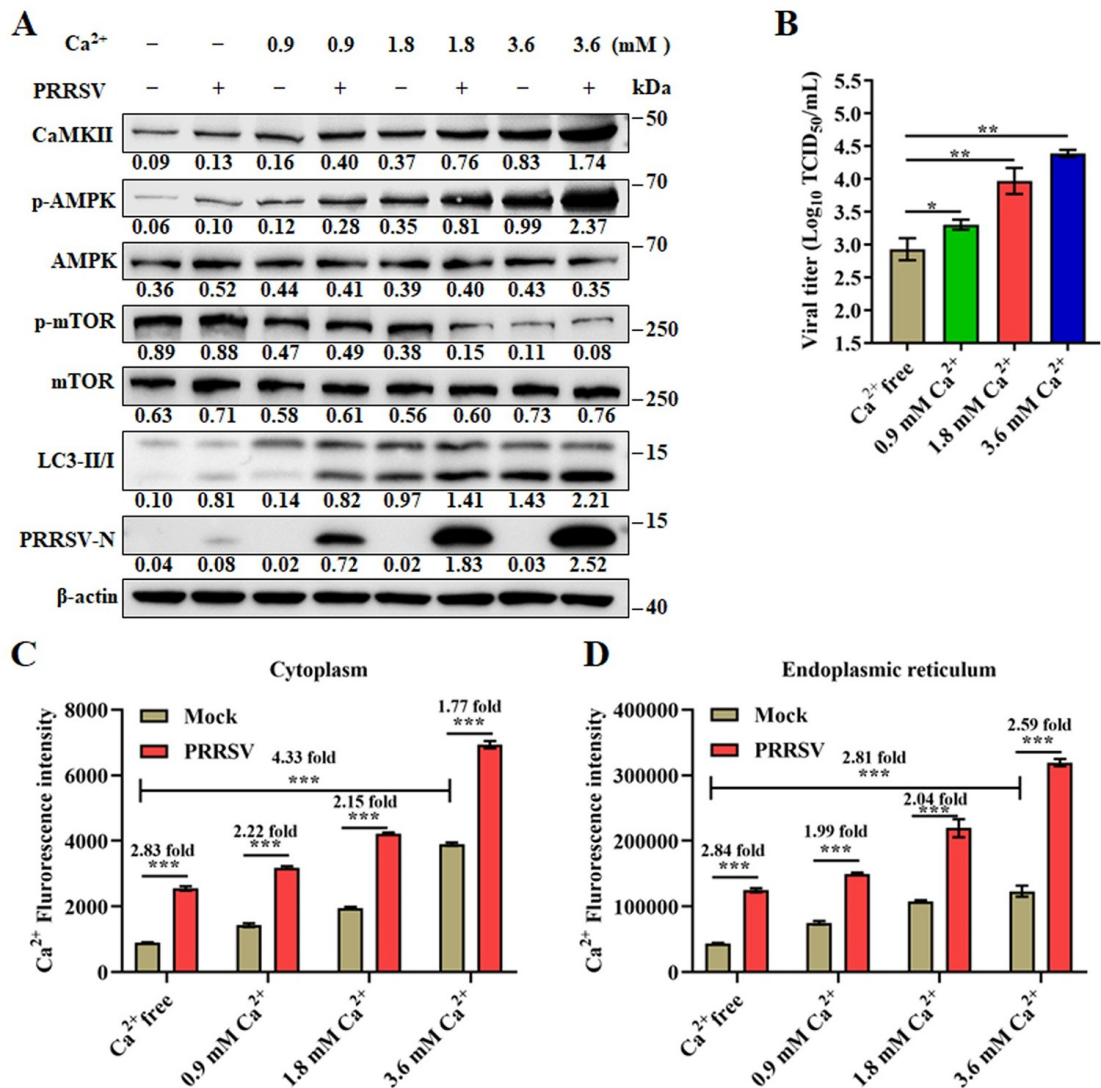
The ER takes up extracellular calcium during PRRSV infection. (A-C) Marc-145 cells were infected with PRRSV (MOI = 0.1) and cultured in media containing 0, 0.9, 1.8, or 3.6 mM calcium chloride for 24 h. (**A**) Cell lysates were analyzed by western blotting for CaMKII, p-AMPK, AMPK, p-mTOR, mTOR, LC3-I/II, PRRSV-N, and β-actin. (**B**) TCID_50_ of PRRSV in cell supernatants. (**C**) Cytoplasmic Ca^2+^ levels were determined by fluorescence of Fluo-8. (**D**) ER associated Ca^2+^ levels were determined by a Fluo-8 calcium flux analysis kit as described in Material and Methods. The protein levels were quantified by Image J and normalized to β-actin. The data are representative of results from three independent experiments. Error bars indicate the mean (± SD) of three independent experiments. *, p < 0.05; **, p < 0.01; and ***, p < 0.001.

At the cellular level, Ca^2+^ is derived from two sources- external and internal [25,26]. As illustrated, PRRSV replication is significantly greater when infected cells are in normal medium *vs* calcium free medium. To further investigate the role of exogenous Ca^2+^ on PRRSV replication, we incubated infected cells in culture media containing various concentrations of Ca^2+^, then determined intracellular Ca^2+^ levels and the corresponding levels of autophagy. We found that autophagy increased as a function of Ca^2+^; the levels of CaMKII increased, the cascade of p-AMPK increased, and the levels of negative feedback p-mTOR decreased (Figs 5A and S7). The viral titers also increased as a function of Ca^2+^ concentration (Fig 5B). As expected, the level of cytosolic and ER Ca^2+^ increased as a function of exogenous Ca^2+^ in both infected and uninfected cells, but the increase in the infected cells was significantly higher than in the uninfected cells (Fig 5C and 5D). These results indicate that PRRSV infection leads to an influx of extracellular Ca^2+^, which favors PRRSV replication by invoking CaMKII-AMPK-mTOR signaling.

**Fig 6:**
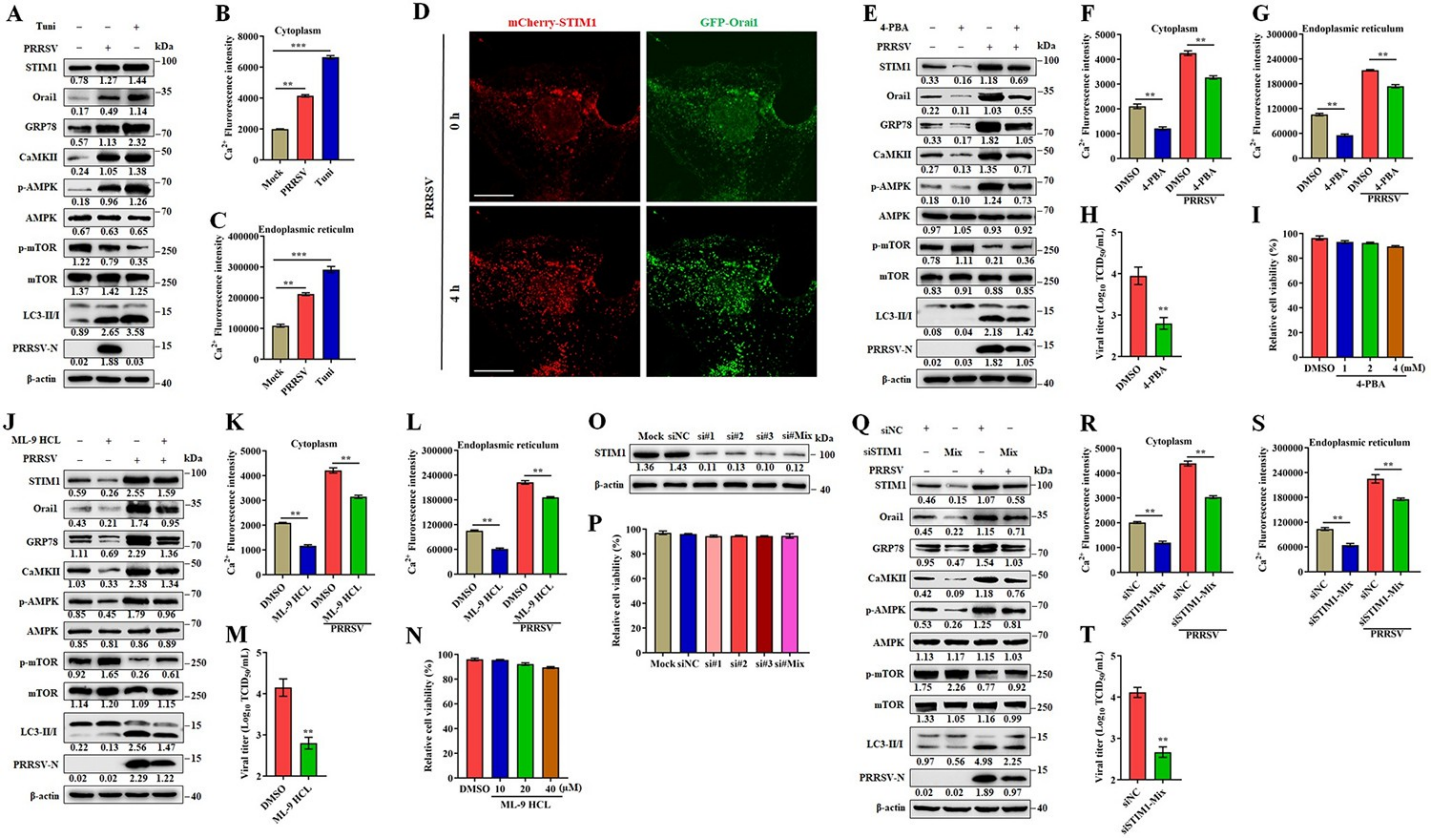
PRRSV infection induces ER stress and SOCE channel take up extracellular calcium. (A-C) Marc-145 cells were infected with PRRSV (MOI = 0.1) or treated with tunicamycin for 24 h. (**A**) Cell lysates were analyzed by western blotting for STIM1, Orai1, GRP78, CaMKII, p-AMPK, AMPK, p-mTOR, mTOR, LC3-I/II, PRRSV-N, and β-actin. (**B**) Cytoplasmic Ca^2+^ and (**C**) ER Ca^2+^ were determined by fluorescence of Fluo-8. (**D**) Marc-145 cells were transfected with mCherry-STIM1 and GFP-Orai1 for 24 hours, followed by infection with PRRSV. mCherry-STIM1 and GFP-Orai1 were visualized by confocal microscopy at indicated timepoints. Scale bar, 5 μm. (E-I) Marc-145 cells were infected with PRRSV (MOI = 0.1) and treated with 4-PBA (2.0 mM) or DMSO for 24 h. (**E**) Cell lysates were analyzed by western blotting for STIM1, Orai1, GRP78, CaMKII, p-AMPK, AMPK, p-mTOR, mTOR, LC3-I/II, PRRSV-N, and β-actin. (**F**) Cytoplasmic Ca^2+^ and (**G**) ER Ca^2+^ were determined by fluorescence of Fluo-8. (**H**) TCID_50_ of PRRSV in cell supernatants. (**I**) The effect of 4-PBA on Marc-145 cell viability. Marc-145 cells were treated with 4-PBA at indicated concentrations or DMSO for 24 h, and then analyzed with CCK-8 system. (J-N) Marc-145 cells were infected with PRRSV (MOI = 0.1) and treated with ML-9 HCL (25 μM) or DMSO for 24 h. (**J**) Cell lysates were analyzed by western blotting for STIM1, Orai1, GRP78, CaMKII, p-AMPK, AMPK, p-mTOR, mTOR, LC3-I/II, PRRSV-N, and β-actin. (**K**) Cytoplasmic Ca^2+^ and (**L**) ER Ca^2+^ were determined. (**M**) TCID_50_ of PRRSV in cell supernatants. (**N**) The effect of ML-9 HCL on Marc-145 cell viability. Marc-145 cells were treated with ML-9 HCL at indicated concentrations or DMSO for 24 h, and then analyzed with CCK-8 system. (O-T) STIM1 is essential for PRRSV-induced Ca^2+^ influx, activation of CaMKII-AMPK-mTOR-LC3II signaling, and PRRSV efficient replication. (**O**) Western blotting was used to quantitate the level of STIM1 in siRNA1-, siRNA2-, siRNA3-, Mix (the equal amounts of siRNA1, siRNA2 and siRNA3) or siNC-transfected Marc-145 cells. (**P**) The cell viability of Marc-145 cells transfected with in siRNA1, siRNA2, siRNA3 or Mix (the equal amounts of siRNA1, siRNA2 and siRNA3) targeting to STIM1, or siNC. (Q-T) Marc-145 cells were transfected with siRNA Mix (the equal amounts of siRNA1, siRNA2 and siRNA3) targeting to STIM1 or siNC for 24 h, and mock or infected with PRRRV (MOI = 0.1) for another 24 h. (**Q**) Cell lysates were prepared and analyzed by immunoblotting using anti-STIM1, anti-Orai1, anti-GRP78, anti-CaMKII, anti-p-AMPK, anti-AMPK, anti-p-mTOR, anti-mTOR, anti-LC3-I/II, anti PRRSV-N, and anti-β-actin antibodies. (**R**) Cytoplasmic Ca^2+^ and (**S**) ER Ca^2+^ were determined. (**T**) TCID_50_ of PRRSV in cell supernatants. The protein levels were quantified by Image J and normalized to β-actin. The data are representative of results from three independent experiments. Error bars indicate the mean (± SD) of three repeats. *, p < 0.05; **, p < 0.01; and ***, p < 0.001.

Because the Ca^2+^ balance is associated with ER stress [14]. We additionally monitored the changes of Ca^2+^ content in the cytoplasm and ER at the early stage of PRRSV infection. We noted that ER associated Ca^2+^ was temporarily decreased at 2–4 hpi, and rapidly increased at later timepoints, while the cytoplasmic Ca^2+^ was continuously increased (S8 Fig). These results imply that the Ca^2+^ influx caused by PRRSV infection is related to ER stress. Calcium channels in the plasma membrane are the mediators of Ca^2+^ entry from the extracellular medium. SOCE channel is the major Ca^2+^ entry pathway in non-excitable cells. Orai1 on the plasma membrane and STIM1 on ER are the proteins responsible for SOCE channel activation, which is associated with ER stress [23]. We hypothesized that exogenous Ca^2+^ enters the cytoplasm or ER during PRRSV infection due to the subsequent induction of ER stress and SOCE channel activation. We first quantitated the induced ER stress (GRP78), SOCE channel elements (Orai1 and STIM1) and calcium signaling (CaMKII-AMPK-mTOR-LC3II) differences in infected vs uninfected cells, tunicamycin treated cells were used as positive control. As shown in Figs 6A and S9A, cells infected with PRRSV had significantly elevated levels of GRP78, as well as cytoplasmic and ER Ca^2+^ (Fig 6B and 6C). Consistently, CaMKII-AMPK-mTOR-LC3II signaling was significantly activated as was SOCE channel (reflected by increased levels of Orai1 and STIM1) (Fig 6A). The ER and PM form junctions crucial to the SOCE channel opening [27,28]. Thus, we evaluated the remodeling of ER–PM contact sites (CSs) during PRRSV infection using fluorescently labeled proteins (mCherry-STIM1 and GFP-Orai1) and immunofluorescence microscopy. As anticipated, at 4 hours post infection, STIM1 and Orai1 were found to redistribute within their respective membranes and appear as “puncta”, indicating the formation of closed ER-PM CSs and the opening of SOCE channels in PRRSV-infected cells (Fig 6D).

To further investigate whether PRRSV induced the Ca^2+^ inflow through SOCE channel, we measured the levels of cytoplasmic and ER Ca^2+^ in cells treated with inhibitors of ER stress and SOCE channel. In infected and uninfected cells, when ER stress was inhibited with 4-PBA (a classic ER stress inhibitor), levels of GRP78, STIM1, and Orai1 were decreased. Additionally, CaMKII-AMPK-mTOR-LC3II signaling (Figs 6E and S9B) and levels of cytoplasmic and ER Ca^2+^ were all decreased (Fig 6F and 6G). Not unexpectedly, 4-PBA treatment significantly reduced the PRRSV replication (Fig 6H). In infected and uninfected cells, when STIM1 was inhibited by ML-9 HCL treatment, the levels of STIM1 and Orai1 decreased and ER stress was also inhibited after blocking SOCE channel, as evidenced by the decreased levels of GRP78 (Figs 6J and S9C). These results indicate that Ca^2+^ influx is closely related to ER stress. As expected, ML-9 HCL also impeded the PRRSV-induced Ca^2+^ influx, activation of CaMKII-AMPK-mTOR-LC3II signaling, and virus replication (Fig 6K, 6L and 6M). Besides, the treatment concentrations of 4-PBA and ML-9 HCL had no effect on the viability of Marc-145 cells (Fig 6I and 6N). To exclude the non-specific effects of inhibitors, the siRNA knockdown assays targeting STIM1 and Orai1 were performed, and then the activation of CaMKII-AMPK-mTOR-LC3II signaling, PRRSV-induced Ca^2+^ influx, and virus replication were assayed. The results showed that all three STIM1 siRNA were efficient (Fig 6O), and had no effects on the cell viability (Fig 6P). Further, using siSTIM1-Mix (equal amounts of each three siRNA targeting STIM1) or a single siSTIM1 to reduce STIM1 expression, the decreased PRRSV-induced Ca^2+^ influx, inhibition of CaMKII-AMPK-mTOR-LC3II signaling, and downregulated virus replication were observed (Figs 6Q–6T, S9D and S10G–S10J). Meanwhile, the similar results were seen by using the Orai1 siRNA treatment (S10A–S10F Fig). In all, these results demonstrate that PRRSV infection induces ER stress and results in the uptake of extracellular calcium by SOCE channel.

### ER associated calcium is released into the cytoplasm via IP3R but not RyR channel

**Fig 7:**
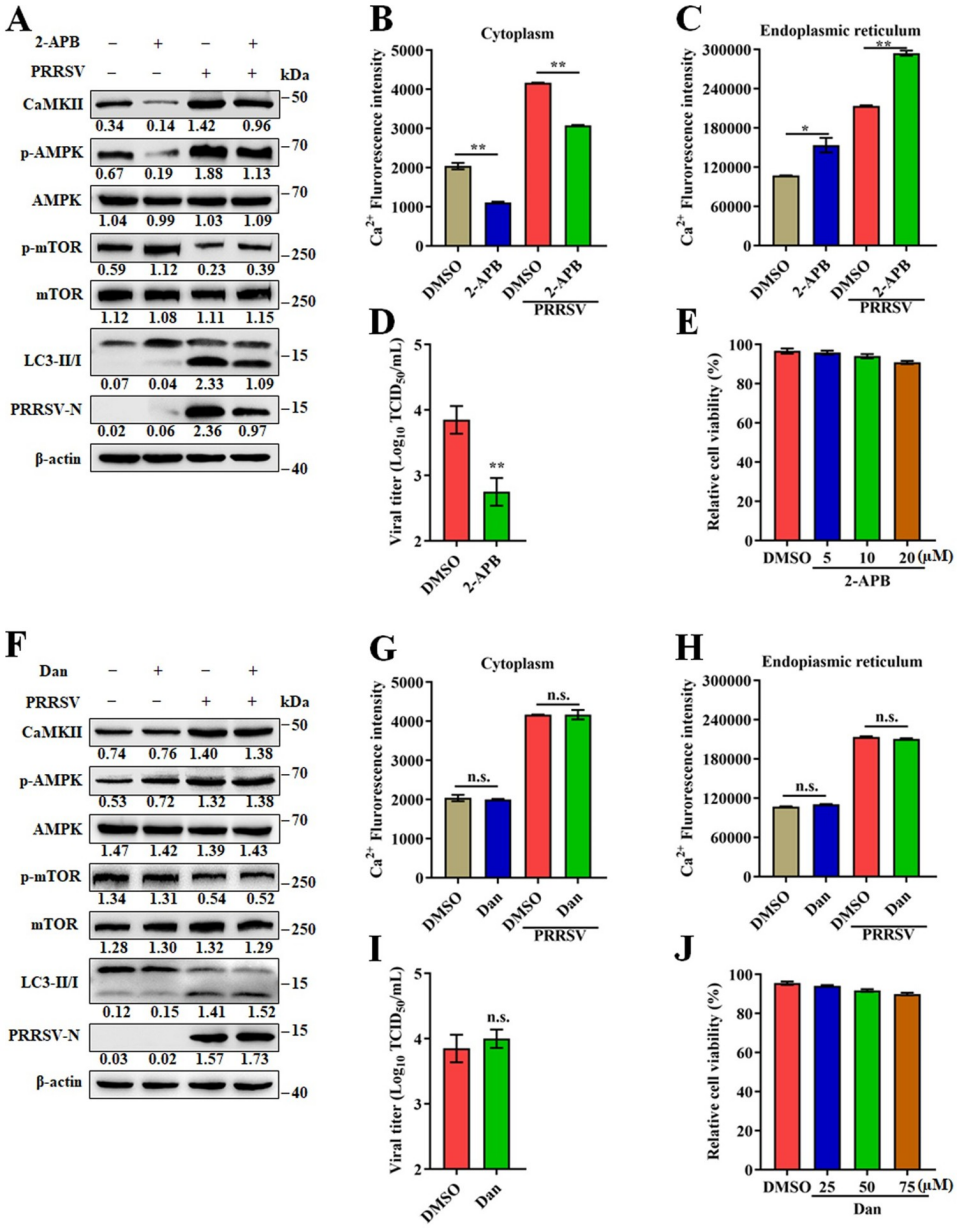
ER associated calcium is released into the cytoplasm from IP3R but not RyR channel. (A-E) Marc-145 cells were infected with PRRSV (MOI = 0.1) and treated with 10 μM 2-APB or DMSO for 24 h. (**A**) Cell lysates were analyzed by western blotting for CaMKII, p-AMPK, AMPK, p-mTOR, mTOR, LC3-I/II, PRRSV-N, and β-actin. (**B**) Cytoplasmic Ca^2+^ levels and (**C**) ER Ca^2+^ levels were determined by fluorescence of Fluo-8. (**D**) TCID_50_ of PRRSV in cell supernatants. (**E**) The effect of 2-APB on Marc-145 cell viability. Marc-145 cells were treated with 2-APB at indicated concentrations or DMSO for 24 h, then analyzed with CCK-8 system. (F-J) Marc-145 cells were infected with PRRSV (MOI = 0.1) and treated with 50 μM dantrolene sodium or DMSO for 24 h. (**F**) Cell lysates were analyzed by western blotting for CaMKII, p-AMPK, AMPK, p-mTOR, mTOR, LC3-I/II, PRRSV-N, and β-actin. (**G**) Cytoplasmic Ca^2+^ levels and (**H**) ER Ca^2+^ levels were determined by fluorescence of Fluo-8. (**I**) TCID_50_ of PRRSV in cell supernatants. (**J**) The effect of Dantrolene sodium on Marc-145 cell viability. Marc-145 cells were treated with Dantrolene sodium at indicated concentrations or DMSO for 24 h, then analyzed with CCK-8 system. The protein levels were quantified by Image J and normalized to β-actin. The data are representative of results from three independent experiments. Error bars indicate the mean (± SD) of three repeats. *, p < 0.05; **, p < 0.01; and ***, p < 0.001.

The ER is an important Ca^2+^ store, but when overloaded it releases Ca^2+^ into the cytoplasm or organelles. ER Ca^2+^-releasing channels, including ryanodine receptor (RyR) and inositol trisphosphate receptor (IP3R), have been functionally and structurally characterized [29]. 2-Aminoethyl Diphenylborinate (2-APB), an IP3 receptor (IP3R) inhibitor, regulates IP3-induced calcium release. Dantrolene sodium acts as a postsynaptic muscle relaxant by antagonizing RyR, resulting in inhibited Ca^2+^ release from sarcoplasmic reticulum stores. When infected and uninfected Marc-145 cells were treated with 2-APB, CaMKII-AMPK-mTOR-LC3II signaling activation was inhibited, cytoplasmic and ER Ca^2+^ levels were significantly reduced (Figs 7A–7C and S11E), and PRRSV replication was significantly inhibited (Fig 7D). Treatment with dantrolene sodium did not affect the replication of PRRSV or the expression of related proteins; Ca^2+^ levels in the cytoplasm and ER were unaffected as well (Figs 7F–7I and S11F). The treatment concentrations of 2-APB and dantrolene sodium had no effect on the viability of Marc-145 cells (Fig 7E and 7J). In addition, other two RyR channel inhibitors, tetracaine HCl and porcaine, also had no effect on PRRSV replication (S11A–S11D Fig). These results suggest that an influx of Ca^2+^ into the ER is released back into the cytoplasm through the IP3R channel not the RyR channel.

### PRRSV Nsp2 interacts with GRP78 and STIM1, induces autophagy

**Fig 8:**
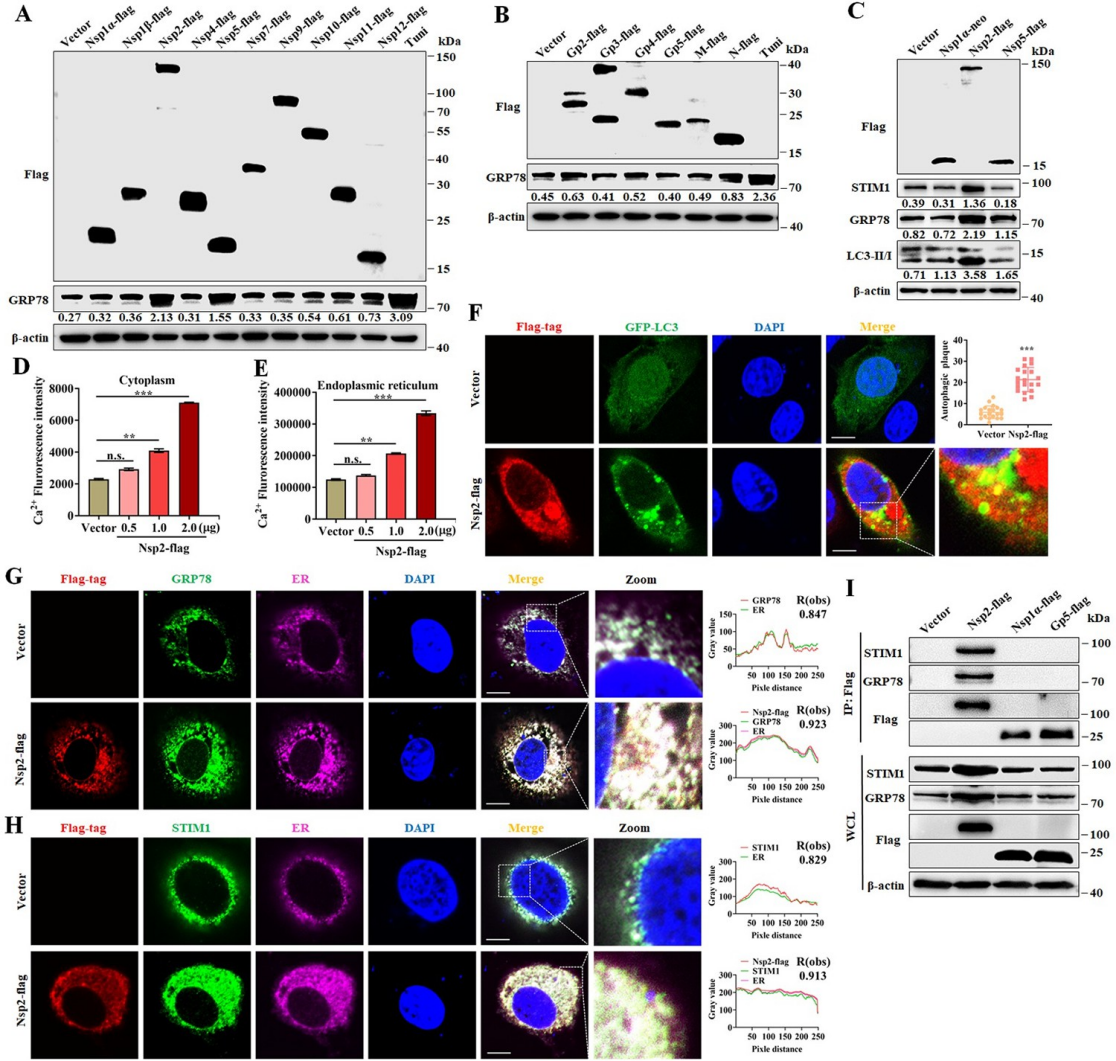
PRRSV Nsp2 interacts with STIM1 and GRP78, induces autophagy. (A and B) The effect of viral non-structural and structural proteins on ER stress. Marc-145 cells were transfected with plasmids encoded non-structural (**A**) and structural (**B**) proteins for 24 h. Cell lysates were harvested for western blotting with antibodies against Flag, GRP78 and β-actin. (C-H) PRRSV Nsp2 induces ER stress and autophagy. (**C**) Marc-145 cells were transfected with pCI-Nsp2-flag or empty vector (pCI-neo) for 24 h then cell lysates were analyzed by western blotting for Flag, STIM1, GRP78, LC3-I/II, and β-actin. (D-E) Marc-145 cells were transfected with pCI-neo or increasing doses of pCI-Nsp2-flag for 24 h. (**D**) Cytoplasmic Ca^2+^ and (**E**) ER Ca^2+^ levels were determined by fluorescence of Fluo-8. (**F**) Marc-145 cells were transfected with pCI-Nsp2-flag or pCI-neo together with GFP-LC3 for 24 h. Cells were stained for Flag, nuclei were stained with DAPI, and cells were observed by confocal microscopy. The numbers of GFP-LC3 dots were quantified from 20 different cells in each group. (**G**) Colocalization of Nsp2 and GRP78 at ER. Cells were stained for Flag, GRP78, Calnexin (ER marker) and nuclei were stained with DAPI; cells were observed by confocal microscopy. Scale bar, 5 μm. (**H**) Colocalization of Nsp2 and STIM1 at ER. Cells were stained for Flag, STIM1, Calnexin (ER marker) and nuclei were stained with DAPI; cells were observed by confocal microscopy. Scale bar, 5 μm. (**I**) PRRSV Nsp2 is associated with GRP78 and STIM1. Marc-145 cells were transfected with pCI-Nsp2-flag, pCI-Nsp1α-flag, pCI-GP5-Flag or pCI-neo for 24 h. Cell lysates were immunoprecipitated with anti-Flag antibody, and then subjected to western blot analysis for Flag, STIM1 GRP78, and β-actin. The protein levels were quantified by Image J and normalized to β-actin. The data are representative of results from three independent experiments. Error bars indicate the mean (± SD) of three repeats. *, p < 0.05; **, p < 0.01; and ***, p < 0.001.

To determine which PRRSV protein is responsible for viral induced ER stress and autophagy, we transfected Marc-145 cells with plasmids encoding the structural and non-structural proteins of PRRSV; cells treated with tunicamycin were the positive control. We found that ER stress was induced in cells overexpressing Nsp2 and Nsp5 (Fig 8A and 8B), however the expression of LC3II and STIM1 was increased only in cells overexpressing Nsp2 (Fig 8C). Consistently, the concentration of Ca^2+^ in cytoplasm and ER increased along with increasing of Nsp2 expression (Fig 8D and 8E), suggesting that Nsp2 plays a dominant role in PRRSV-induced Ca^2+^ influx. Confocal results also showed autophagy induced by Nsp2 (Fig 8F). Confocal experiments showed that Nsp2 interacted with GRP78 and STIM1, and colocalized at ER (Fig 8G and 8H). Further, GRP78 and STIM1 were pulled down by Nsp2 but not Nsp1α or GP5 (also a transmembrane protein) in immunoprecipitation assay (Fig 8I). The kinetics of Nsp2 expression was also determined during PRRSV infection. We found that the Nsp2 started to express at 4 hpi, and steadily increased at later timepoints. The expressions of STIM1, GRP78 and LC3II was also increased in the same manner (S12 Fig). These results suggest that Nsp2 is the viral factor responsible for inducing ER stress and opening SOCE channel during PRRSV infection.

### PRRSV infection triggers autophagy via ER stress-induced calcium signaling in primary cells

**Fig 9:**
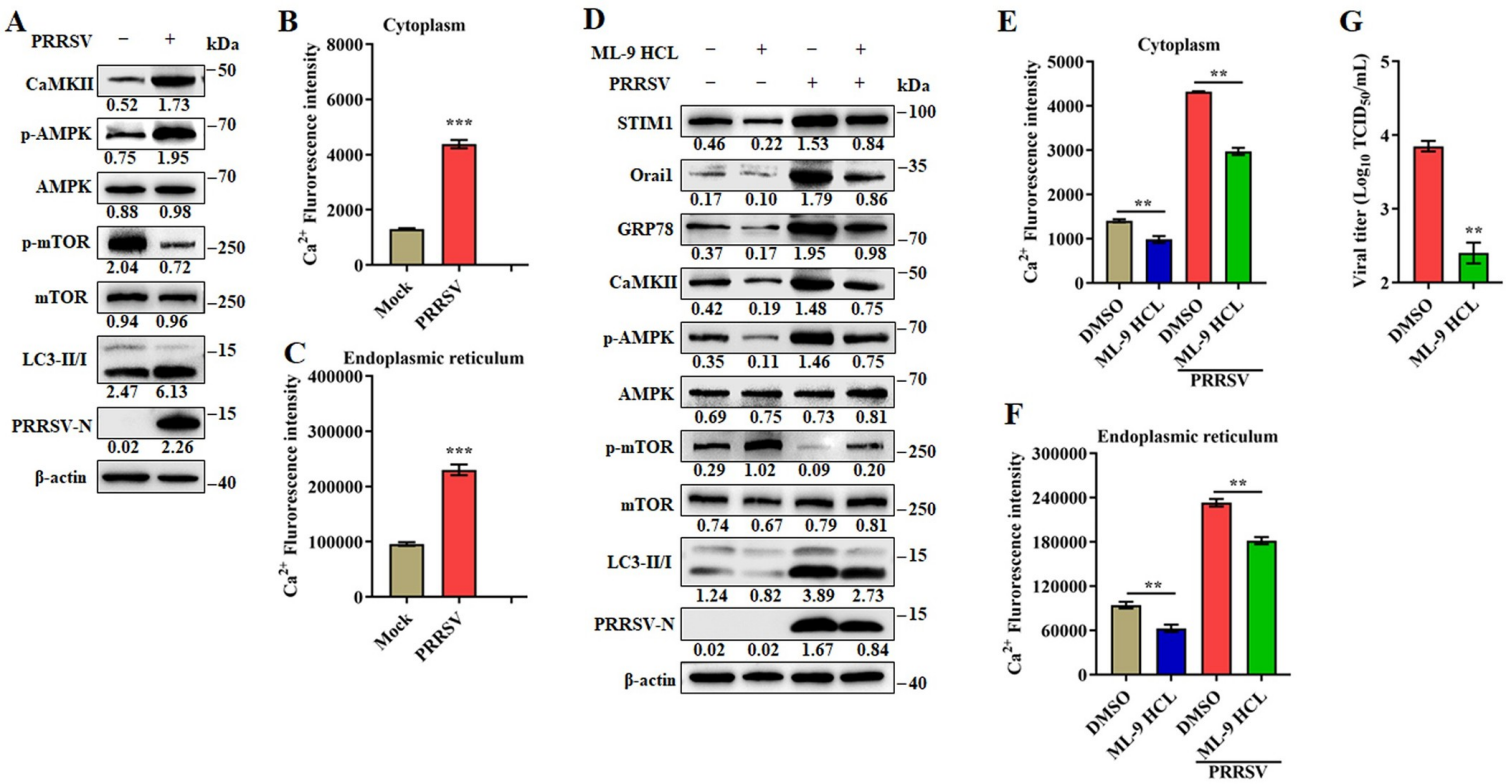
Ca^2+^ promotes PRRSV replication in pulmonary alveolar macrophages (PAMs). (A-C) PAMs were infected with PRRSV (MOI = 0.1) for 24 h. (**A**) Cell lysates were analyzed by western blotting for CaMKII, p-AMPK, AMPK, p-mTOR, mTOR, LC3-I/II, PRRSV-N, and β-actin. (**B**) Cytoplasmic and (**C**) ER Ca^2+^ levels were determined by fluorescence of Fluo-8. (D-G) PAMs were infected with PRRSV (MOI = 0.1) and treated with 25 μM ML-9 HCL or DMSO for 24 h. (**D**) Cell lysates were analyzed by western blotting for STIM1, Orai1, CaMKII, p-AMPK, AMPK, p-mTOR, mTOR, LC3-I/II, PRRSV-N, and β-actin. (**E**) Cytoplasmic and (**F**) ER Ca^2+^ levels were determined by fluorescence of Fluo-8. (**G**) TCID_50_ of PRRSV in cell supernatants. The protein levels were quantified by Image J and normalized to β-actin. The data are representative of results from three independent experiments. Error bars indicate the mean (± SD) of three repeats. *, p < 0.05; **, p < 0.01; and ***, p < 0.001.

Because pulmonary alveolar macrophages (PAMs) are the target cells of PRRSV *in vivo*, we next investigated PRRSV induced ER stress in PAMs. Results showed that, consistent with Marc-145 cells, PRRSV infection initiated CaMKII-AMPK-mTOR-LC3II signaling (Figs 9A and S13A) and levels of cytoplasmic and ER Ca^2+^ increased (Fig 9B and 9C). Further, the STIM1 inhibitor ML-9 HCL, inhibited PRRSV-induced ER stress, CaMKII-AMPK-mTOR-LC3II signaling activation, Ca^2+^ flux, and virus replication in PAMs (Figs 9D–9G and S13B). These effects were tested using two other PRRSV strains, S1 and FJ1401, representative of classic and NADC-30 like strains respectively. The results showed that S1 and FJ1401 infection also resulted in increased cytoplasmic and ER associated Ca^2+^, similar to BB0907. All three strains induced ER stress, Ca^2+^ flux in the ER, and activated the CaMKII-AMPK-mTOR-LC3II pathway to the same statistical extent (S14 Fig). Taken together, these results demonstrate that autophagy induced by ER stress associated Ca^2+^ favors PRRSV replication in PAMs.

## Discussion

Many viruses disturb cellular Ca^2+^ homeostasis by hijacking host calcium channels or pumps, generally benefiting viral replication [23]. In this study, we showed that PRRSV infection caused an influx of extracellular Ca^2+^ and activated autophagy through the CaMKII-AMPK-mTOR-LC3II pathway to the benefit of its replication. This is the first report to link Ca^2+^, autophagy, and PRRSV replication.

Autophagy can support or hinder viral replication depending on the viruses, cell types, or cellular environments [19]. Indeed, many positive strand RNA viruses including picornaviruses and flaviviruses induce the autophagic process during their replicative life cycles generating the membranes necessary for the biogenesis of their replication organelles [19]. Our results demonstrate that autophagy promotes PRRSV replication, which is consistent with previous studies[21].

Ca^2+^ and calcium-sensitive proteins play important roles in the regulation of autophagy. CaMKII is a versatile Ca^2+^-dependent kinase and responds to changes of intracellular Ca^2+^ [30–33]. Porcine circovirus type 2 (PCV2) infection increases cytosolic Ca^2+^ which upregulates CaMKII, CaMKII in turn activates AMPK and calcium/calmodulin-dependent protein kinase I (CaMKI). PCV2 employs CaMKI and Trp-Asp (WD) repeat domain phosphoinositide-interacting protein 1 (WIPI1) as an additional pathway activating AMPK signaling in autophagy initiation [34]. In cells infected with PRRSV, CaMKII expression increased in response to increasing cytoplasmic calcium. CaMKII phosphorylated its downstream effector AMPK, and elevated lipidation of LC3II. The interplay between viruses and Ca^2+^ is dependent on the function of the endoplasmic reticulum [35]. To determine how PRRSV induces Ca^2+^ influx, we first show that PRRSV infection causes an increase in Ca^2+^ in the ER. Previous studies have shown that PRRSV induces ER stress which facilitates virus replication, however, the activation pathways and the biological significance are not clear [36]. We demonstrated that PRRSV induced ER stress serves to open SOCE channel, which allow extracellular Ca^2+^ entry into the intracellular space. Increased intracellular Ca^2+^ induced autophagy through the CaMKII-AMPK-mTOR pathway, thereby benefiting PRRSV replication. These effects are consistent with infection by classical swine fever virus (CSFV) [37].

SOCE channel is major calcium channels on the cell plasma membrane that mediates the entry of Ca^2+^ from the extracellular medium [28,38]. SOCE channel often triggers sustained Ca^2+^ entry across the plasma membrane and tightly link intracellular Ca^2+^ release with Ca^2+^ influx providing a means to refill the Ca^2+^ stores [35,39]. Orai1 on the plasma membrane and STIM1 on ER are the proteins responsible for SOCE channel activation. The depletion of ER Ca^2+^ stores promotes STIM1 protein aggregation and interaction with Orai1 to open the channel, mediating Ca^2+^ entry [28,38]. SOCE channel opening also requires changes of ER-PM CSs [27,28]. Our results of TEM showed that ER is held in close apposition to PM after PRRSV infection, contributing the opening of SOCE channel. We found that Nsp2 plays a dominant role in the PRRSV induced continuous inflow Ca^2+^ through the SOCE channel. Taken in combination with the kinetics of Nsp2 expression and ER associated Ca^2+^, we assume that PRRSV infection consumes ER associated Ca^2+^ before Nsp2 expression, and the ER takes up extracellular Ca^2+^ via SOCE channel after Nsp2 expression. Nsp2 has been reported to play a role in inducing autophagy [40], and our findings illustrate a possible mechanism. Notably, we observed that overexpression of Nsp2 tagged by Flag could induce ER stress by increasing GRP78. In contrast, Catanzaro et al. demonstrated that EGFP-Nsp2 transfection could not promote GRP78 protein expression [41]. This discrepancy might result from different tagging strategies. When we designed our tagged Nsp, we considered the potential impact of a large tag (e.g., EGFP) on the distribution of Nsp and thus tagged Nsp with a short Flag tag. Our data clearly show that Flag-Nsp2 colocalized with ER markers (Fig 8G and 8H). Thus, one possible explanation for the attenuation of ER stress in the previous report might result from the mislocation of EGFP-Nsp outside the ER. Evidence has emerged that pharmacologically targeting calcium channels or calcium release from the ER can obstruct virus lifecycles, and impeding viral-induced dysregulated Ca^2+^ homeostasis is a potential strategy in the development of antiviral drugs [23]. Our evidence shows that 4-PBA can inhibit PRRSV replication by inhibiting ER activation, and ML-9 HCL can inhibit virus replication by inhibiting SOCE channel. Furthermore, our findings demonstrate that BAPTA-AM reduces the biosynthesis of viral sub-genomic RNA, suggesting the crucial role of BAPTA-AM in the formation of replication organelles (ROs) during PRRSV replication. Importantly, Nsp2 and Nsp5 are key constituents of viral ROs [42]. Thus, research regarding the functions of Nsp2 and Nsp5 in ER membrane remodeling, viral ROs formation and Ca^2+^ signals alteration is warranted. In all, we described the positive roles of cytosolic or ER Ca^2+^ in PRRSV replication, in which the channels of SOCE and IP3R were involved. However, the roles of other channels or pumps, such as Sec61 complex and SERCA, in the process of PRRSV replication are warranted [43,44]. In addition, lysosome is also an important Ca^2+^ reservoir [45], which deserves to be further defined in PRRSV infection.

**Fig 10:**
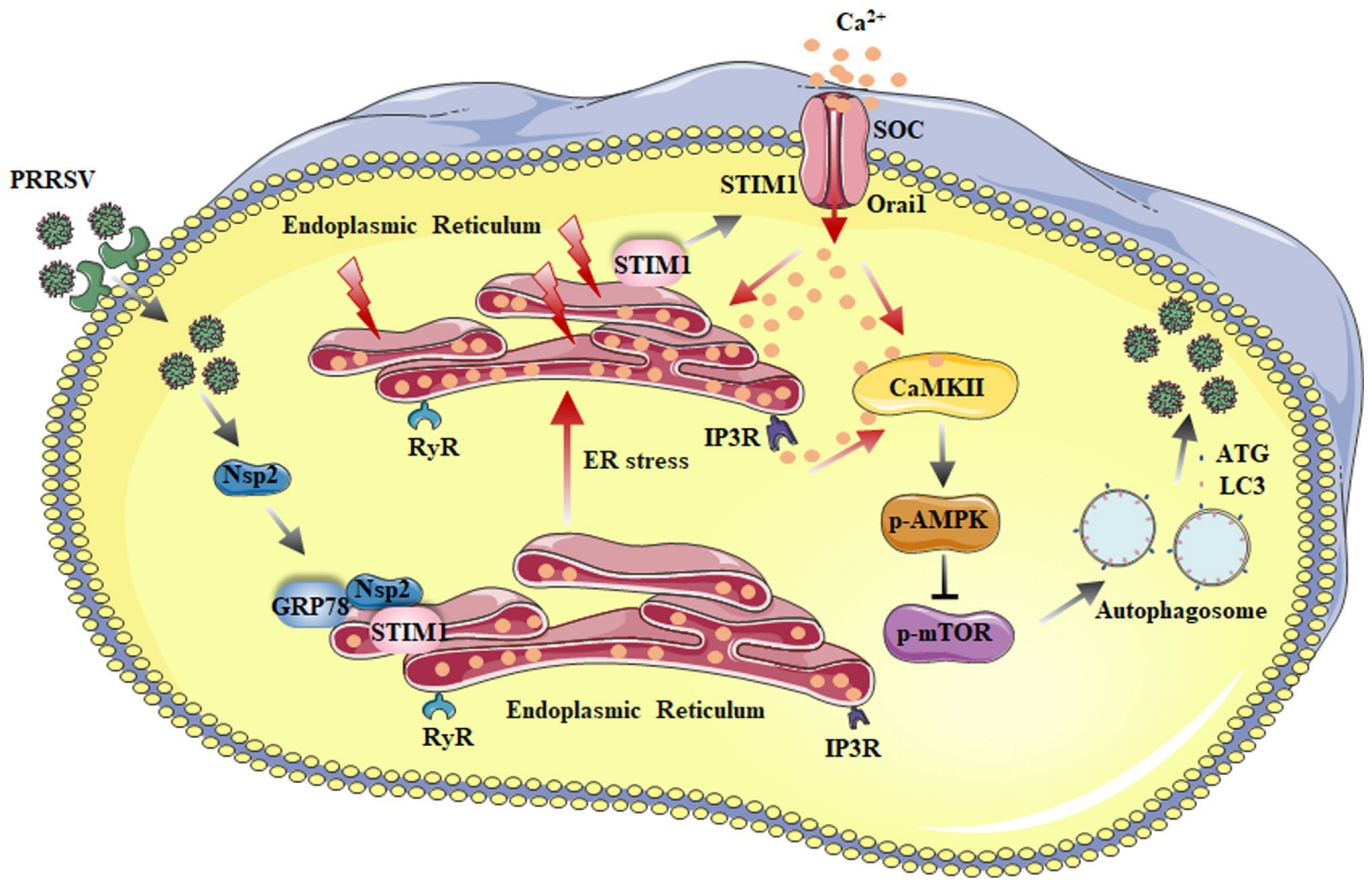
Model of PRRSV inducing autophagy via ER stress-induced calcium signaling pathway. PRRSV attaches to and enters cells, followed by viral protein Nsp2 interacting with GRP78 and STIM1 and inducing ER stress. ER stress results in the STIM1 moving towards to the cell membrane, opening the SOCE channel, inducing Ca^2+^ influx, and finally activating the CaMKII-AMPK-mTOR-autophagy pathway.

In conclusion, we demonstrate the following actions upon PRRSV infection: i) Nsp2 binds with GRP78 and STIM1 inducing ER stress which results in opening of SOCE channel allowing the influx of Ca^2+^ into the ER or cytoplasm; ii) excess ER Ca^2+^ is released into the cytoplasm via IP3R channel; iii) elevated levels of Ca^2+^ in the cytoplasm activate the CaMKII-AMPK-mTOR autophagy pathway, which in turn is beneficial to viral replication (Fig 10). These results expand our understanding of the PRRSV infection mechanism and provide insight into alternative strategies for viral disease control.

## Materials and methods

### Ethics statement

All animal experiments conformed to the rules of National Guidelines for Housing and Care of Laboratory Animals (China) and were performed after obtaining the approval of the Institutional Animal Care and Ethics Committee of Nanjing Agricultural University (permit no. IACECNAU20191002).

### Cells and viruses

Marc-145 cells was obtained from the American Type Culture Collection and cultured in Dulbecco’s modified Eagle’s medium (DMEM; Invitrogen, USA) containing 10% fetal bovine serum (FBS; Gibco, USA), 100 U/ml penicillin, and 100 μg/ml streptomycin. This medium was also used for PRRSV propagation and titration. Pulmonary alveolar macrophages (PAMs) were collected from 4-week-old, specific-pathogen-free piglets as previously described [46]. These cells were maintained in RPMI 1640 medium containing 10% FBS, 100 U/ml penicillin, and 100 μg/ml streptomycin. All cells were kept in a humidified 5% CO_2_ incubator at 37°C. North American genotype 2 PRRSV strains were used for this investigation; the highly pathogenic PRRSV strain BB0907 (GenBank accession no. HQ315835.1) is referred to as PRRSV. PPRSV-GFP is a recombinant virus rescued from an infectious cDNA clone of BB0907 [47], in which the GFP was inserted into and fused with Nsp2. The PRRSV strains S1 (GenBank accession no. DQ459471.1) and FJ1402 (GenBank accession no. KX169191.1) are specifically mentioned by the name (S1, a classical strain; FJ1402, a NADC30-like strain).

### Antibodies and chemicals

Primary antibodies were mouse anti-PRRSV N protein (produced in our laboratory), mouse anti-β-actin (Proteintech, #A5316), mouse anti-Flag (Proteintech, #80010-1-RR), rabbit anti-LC3 (Proteintech, #14600-1-AP), rabbit anti-ATG7 (Cell Signaling Technology, #2631), rabbit anti-CaMKII (Abcam, #ab168818), rabbit anti-p-AMPK (Thr172) (Cell Signaling Technology, #2535), rabbit anti-AMPK (Cell Signaling Technology, #2532), rabbit anti-p-mTOR(Ser2448) (Cell Signaling Technology, #5536), rabbit anti-mTOR (Cell Signaling Technology, #2983), rabbit anti-STIM1 (Cell Signaling Technology, #4916), mouse anti-Orai1 (Proteintech, #66223–1), rabbit anti-GRP78 (Proteintech, #11587-1-AP) and rabbit anti-Calnexin (Abcam, #ab92573). HRP-labeled rabbit or mouse secondary antibodies were purchased from Beyotime (China).

Chemicals used included 2-Aminoethyl diphenylborinate (2-APB) (Selleck, #S6657), 3-MA (Selleck, #S2767), 4-Phenylbutyric acid (4-PBA) (Selleck, #S3592), BAPTA-AM (Selleck, #S7534), Dantrolene sodium (Selleck, #S5478), Dorsomorphin (Compound C) 2HCL (Selleck, #S7306), KN-93 (Selleck, #S7423), ML-9 HCL (Selleck, #S6847), Procaine (Selleck, #S4668), Rapamycin (MedChemExpress, #HY-10219), Tetracaine-HCL (Selleck, #S2573), Thapsigargin (Selleck, #S7895), Tunicamycin (Beyotime, #SC0393).

### Plasmid construction and transfection

Expression plasmids encoding PRRSV proteins with C-terminal Flag tags were constructed by RT-PCR amplification from the genomic RNA of PRRSV strain BB0907 as previously described [47,48]. All viral encoded proteins were cloned into pCI-neo plasmids, and confirmed by DNA sequencing. The Orai1 gene (GenBank accession no. XM_001095850.4) was amplified from Macaca mulatta Marc-145 cells using reverse transcription-PCR (RT-PCR) and inserted into the lentivector pCDH-EF1α-MCS-IRES-GFP (Systembio, USA) to create pCDH-GFP-Orai1 (GFP-Orai1). The STIM1 gene (GenBank accession no. NM_001261535.1) was amplified from Marc-145 cells and fused with the mCherry gene before being inserted into pCAGGS, resulting in pCAGGS-mCherry-STIM1 (mCherry-STIM1). Both constructs were verified by DNA Sanger sequencing. Transfection with these expression plasmids were done in Marc-145 cells using Lipofectamine 3000 (Thermo Fisher, USA) according to the manufacturer’s instructions.

### TCID_50_ determination

Viral titers were determined by TCID_50_ assay. Briefly, Marc-145 cells were seeded into 96-well plates at 1×10^4^ cells per well and incubated overnight. Cells were inoculated with 100 μL of 10-fold serially diluted virus and incubated for 1 h at 37°C. The inoculum was then removed, and cells were washed with PBS. Maintenance medium (DMEM containing 2% FBS) was added to each well and the cells were cultured for 3 to 5 days. Cells were observed daily for CPE and the TCID_50_ was calculated by the Reed-Muench method [49].

### Ca^2+^ measurements

Cytosolic Ca^2+^ was measured as previously described with minor modifications [27]. A Fluo-8 Calcium Flux Analysis Kit (Abcam, ab112129) was used to measure cytoplasmic Ca^2+^. Briefly, at indicated timepoints, 100 μL of Fluo-8 dye-loading solution was aliquoted into wells of a 96-well plate containing 1×10^4^ cells per well and incubated for 30 minutes at 37°C then another 30 minutes at room temperature. Calcium flux was determined by measuring fluorescence at Ex/Em = 490/525 nm.

To visualize the correlation between PRRSV infection and cytoplasmic Ca^2+^, the cells were infected with PRRSV-GFP and the Ca^2+^ concentration was measured by Rhod-2 (R1245, Thermo Fisher, USA) as previously described [50]. Briefly, Marc-145 cells were infected with PRRSV-GFP at indicated MOIs or timepoints, then the cells were incubated with Rhod-2 at a concentration of 5μM. After incubation at 37°C in the dark incubator for 30 min, the cells were observed with a fluorescence microscope.

ER associated Ca^2+^ measurement was performed as described elsewhere. TG inhibits Ca^2+^-ATPase in the endoplasmic reticulum. Ca^2+^ is released into the cytoplasm via channels in the ER, while the Ca^2+^-ATPase in the ER pumps it back from the cytoplasm. When the ATPase pump in the ER is inhibited by TG, Ca^2+^ that leaks from the ER is not re-sequestered by the pumps, and Ca^2+^ accumulates in the cytosol. In cells treated with TG, the Ca^2+^ level detected in the cytosol is thought to represent the concentration of Ca^2+^ in the ER [51–53]. Briefly, Marc-145 cells were washed twice in Ca^2+^-free HBSS and suspended in Ca^2+^-free HBSS. To measure the Ca^2+^ in ER, 1 μM (final concentration) TG was added to the Ca^2+^-free HBSS immediately, and the Ca^2+^ was measured as described above.

### Cell viability assay

Cells viability was determined using CCK-8 assays. Briefly, Marc-145 cells were seeded into 96-well plates and treated with either Ca^2+^ free media, BAPTA-AM, N-93, Compound C, 4-PBA, ML-9 HCL, 2-APB, Dan, Tetra, Porcaine, siCaMKII, siSTIM1 or siOrai1 at indicated doses for 24 h. 10 μL CCK-8 solution (Beyotime, China) was added to each well. After 2 h of incubation, the optical density of each well was measured at a wavelength of 450 nm.

### Quantification of PRRSV vRNA by qRT-PCR

Cells were harvested and total RNA was extracted from PRRV-infected cells by using the Total RNA Kit I (Omega Bio-tek). RNA was then reverse transcribed using a HiScript II 1st Strand cDNA Synthesis Kit (Vazyme, China), according to the manufacturer’s instructions. qRT-PCR was performed with the AceQ qPCR SYBR Green Master Mix (Vazyme, China). The following qRT-PCR primers were used: PRRSV-ORF7-Fwd: 5’-AAA CCA GTC CAG AGG CAA G-3’; PRRSV-ORF7-Rev: 5’-TCA GTC GCA AGA GGG AAA T-3’ and GAPDH-Fwd: 5’-GAA GGT GAA GGT CGG AGT C-3’; GAPDH- Rev: 5’- GAA GAT GGT GAT GGG ATT TC-3’.

### PRRSV binding and internalization assays

The assays for PRRSV binding and internalization were determined as described elsewhere [54]. For virus binding assay, Marc-145 cells were incubated with BAPTA-AM (50μM)/DMSO or Ca^2+^ free/Ca^2+^ medium and PRRSV for 1 h at 4°C. The cells were washed with ice-cold PBS and harvested, and then the mRNA levels of PRRSV-ORF7 were measured by using qRT-PCR. For virus internalization assay, Marc-145 cells were incubated with PRRSV (1 MOI) for 1 h at 4°C. Afterwards, cells were washed 3 times with ice-cold PBS to remove unbound virus. Then, the culture medium was replaced with fresh DMEM containing BAPTA-AM (50μM)/DMSO or Ca^2+^ free/Ca^2+^ medium, and the cells were incubated for 1 h at 37°C. Cells were washed with citrate buffer (pH 3) to remove non-internalized virus. The levels of PRRSV-ORF7 mRNA were detected by qRT-PCR.

### Western blotting

RIPA buffer (20 mM Tris (pH7.5), 150 mM NaCl, 1% Triton X-100, sodium pyrophosphate, β-glycerophosphate, EDTA, Na_3_VO_4_, and leupeptin) (Beyotime, China) was used to extract total cell protein. Protein concentrations were determined by BCA assay according to the manufacturer’s instructions (Beyotime, China). After boiling and denaturing, 30 μg of protein was separated by 10% SDS-PAGE and transferred to PVDF film (EMD Millipore, USA). Membranes were blocked with TBS-T (50 mM Tris-HCl, pH 7.6, 150 mM NaCl, and 0.1% Tween-20) containing 5% skim milk for 2 h at room temperature. Membranes were incubated with primary antibodies overnight at 4°C. Membranes were washed three times for 10 min with TBS-T, then incubated with HRP-labeled anti-rabbit or anti-mouse antibodies for 1 h at room temperature. Membranes were washed three times for 10 min with TBS-T then bands were visualized using ECL reagent and a gel imaging system (ImageQuant LAS 500; Cytiva). Bands were quantitated using ImageJ 1.8.0 software (National Institutes of Health).

### Immunoprecipitation

Marc-145 cells were transfected with Flag-tagged *Nsp2*, *Nsp1α* or *GP5* using Lipofectamine 3000 (Invitrogen) according to the manufacturer’s instruction for 24 h. Cells were washed with cold PBS and lysed with RIPA buffer for western blotting and immunoprecipitation. Briefly, cell lysates were centrifuged at 12,000 × *g* for 10 min, supernatants were collected and incubated with anti-Flag M2 affinity gel (Sigma-Aldrich, # A2220) for 8 h at 4°C. Beads were collected by centrifugation at 2,500 × *g* for 5 min and washed five times with cold PBS. Bound proteins were subjected to SDS-PAGE, transferred to nitrocellulose, and probed with indicated primary antibodies.

### Confocal microscopy

To detect autophagy induced by PRRSV infection, mCherry-GFP-LC3 plasmid (Addgene #110060) was transfected into Marc-145 cells for 16 h, followed by infection with PRRSV or treatment with drugs for 24 h. The cells were fixed with 4% paraformaldehyde and nuclei were stained with DAPI (Invitrogen, USA) for 15 min at room temperature.

To detect autophagy induced by Nsp2, cells were transfected GFP-LC3 (Addgene, #11546) together with *Nsp2* or vector for 24 h. Cells were fixed with 4% paraformaldehyde in PBS for 30 min at 4°C. After 3 washes with ice-cold PBS, the cells were permeabilized with 0.1% Triton X-100 for 15 min and then blocked in 5% bovine serum albumin (BSA) in PBS for 1 h at 37°C. Cells were then incubated with appropriate primary antibodies (mouse anti-FLAG) for 1 h at 37°C. Cells were washed with PBS and then incubated with Alexa Fluor 594-conjugated goat anti-mouse IgG (H + L) (Thermo Fisher, USA) for 1 h at 37°C in the dark and then stained with DAPI.

For observation of co-localization of Nsp2, STIM1 or GRP78 and ER (Calnexin). Before that, the stained antibodies were prepared as following: rabbit anti-GRP78/STIM1 polyclonal antibody is combined with FITC dye (Yeasen, 60514ES60) according to the manufacturer’s instructions. Rabbit anti-Calnexin monoclonal antibody binds to Cy5 (Meilunstar, MB12193). Afterward, Marc-145 cells were transfected with Flag-tagged Nsp2 or vector for 24 h. Cells were then incubated with antibodies (mouse anti-FLAG plus rabbit anti-GRP78-FITC or rabbit anti-STIM1-FITC and anti-Calnexin-Cy5) for 1 h at 37°C. Cells were washed with PBS and incubated with Alexa Fluor 594-conjugated goat anti-mouse IgG (H + L) (Thermo Fisher, USA) for 1 h at 37°C in the dark, then stained with DAPI. Fluorescence was observed by confocal microscopy (LSM800, Zeiss).

### Live cell imaging of aggregation of STIM1

STIM1 aggregation and recruitment to the plasma membrane were observed as previously described with minor modifications [55,56]. Briefly, Marc-145 cells were seeded in 22 mm confocal dishes, when confluency of cells reached to 60–70%, the transfection complex mix, containing transfection reagent and pCDH-GFP-Orai1/mCherry-STIM1 plasmid DNA, was added. After 24 hours, the cells were infected with PRRSV, and confocal dishes were placed in the incubation chamber of a Nikon confocal microscope at the indicated timepoints. Picture capture was performed, with the excitation light intensity adjusted to 10–20% of maximum intensity to prevent photo-bleaching. High magnification imaging (60× oil) was then performed using the same protocol.

### Transmission electron microscopy (TEM)

Marc-145 cells were mock infected or infected with PRRSV at an MOI 0.1 for 24 h. Then, the cells were harvested and then fixed with 2.5% glutaraldehyde at 4°C for 12 h, post-fixed in 1% osmium tetroxide, dehydrated in graded ethanol, and embedded in epoxy resin. Next, ultrathin sections were stained with uranyl acetate and lead citrate. Finally, the autophagosome-like vesicles were observed on a Hitachi TEM HT7700 (Hitachi, Tokyo, Japan).

### siRNA knock down

The siATG7 (sense strand: 5’-GCU CUU CCU UAC UUC UUA A-3’, and antisense strand: 5’-UUA AGA AGU AAG GAA GAG C-3’), siCaMKII (#1, sense strand: 5’-GGA UCA CCA GAA ACU AGA A-3’ and antisense strand: 5’-UUC UAG UUU CUG GUG AUC C-3’; #2, sense strand: 5’-CAG AGA GUA CUA CAG UGA A -3’ and antisense strand: 5’-UUC ACU GUA GUA CUC UCU G-3’; #3, sense strand: 5’-GAG UGU UUG CGC AAG UUC A-3’ and antisense strand: 5’- UGA ACU UGC GCA AAC ACU C-3’;), siSTIM1 (#1, sense strand: 5’-GGA UGA UGU AGA UCA UAA ATT-3’ and antisense strand: 5’-UUU AUG AUC UAC AUC AUC CTT-3’; #2, sense strand: 5’-GAG GUG CAA UAU UAC AAC ATT -3’ and antisense strand: 5’-UGU UGU AAU AUU GCA CCU CTT-3’; #3, sense strand: 5’-GUC ACC AAC ACC ACA AUG ATT-3’ and antisense strand: 5’-UCA UUG UGG UGU UGG UGA CTT-3’;), siOrai1 (sense strand: 5’-CCU UCG GCC UGA UCU UUA UTT-3’, and antisense strand: 5’-AUA AAG AUC AGG CCG AAG GTT-3’) or siNC (sense strand: 5’-UUC UCC GAA CGU GUC ACG U-3’ and antisense strand: 5’- ACG UGA CAC GUU CGG AGA A-3’) were transfected into Marc-145 cells using Lipofectamine RNAiMAX (Thermo Fisher) for 24 h then infected with PRRSV at an MOI of 0.1 and incubated for 1 h 37°C. Cells were washed and cultured at the indicated conditions, then collected for western blotting.

### Data analysis

All data were analyzed using GraphPad Prism 7.0 software (GraphPad Software, Inc.) and are presented as means ± SD. The intensities of the western blot bands were analyzed using ImageJ 1.8.0 software (National Institutes of Health). Student’s *t*-test was used to compare differences between two groups for normally distributed data. A one-way ANOVA with Dunnett’s test was used to compare differences between three groups. A two-way ANOVA with Tukey’s or Sidak’s multiple-comparisons test was used to evaluate experiments involving multiple groups. The p values were calculated from three biological replicates unless otherwise indicated in the legends. Data were reproduced in independent experiments as indicated in the legends.

## Supporting information

S1 Fig Infection with PRRSV results in increased cytoplasmic Ca^2+^. (A-B) Marc-145 cells were infected at MOI of 0.1 for different timepoints (6, 12, 24, 36 h). (**A**) PRRSV growth kinetics. (**B**) Increase in cytoplasmic Ca^2+^ over infection time course. (C-D) Marc-145 cells were infected with PRRSV at different MOIs for 24 h. (**C**) Viral titers as a function of MOI at 24 hpi. (**D**) Cytoplasmic Ca^2+^ as a function of MOI. Data are expressed as means ± SD (n = 3). *p<0.05; **p < 0.01; ***p < 0.001.
(PDF)

S2 Fig Ca^2+^ is not associated with PRRSV entry. (A-D) The effect of BAPTA-AM or Ca^2+^ concentration on PRRSV adsorption and internalization. (**A and C**) Adsorption assay. Cells were incubated with a mixture of BAPTA-AM (50μM)/DMSO (**A**) or Ca^2+^ free/Ca^2+^ medium (**C**) and PRRSV for 1 h at 4°C and then harvested for qRT-PCR. (**B and D**) Internalization assay. Cells were incubated with PRRSV (1 MOI) for 1 h at 4°C, washed, and finally incubated with BAPTA-AM (50μM)/DMSO (**B**) or Ca^2+^ free/Ca^2+^ medium (**D**) for another 1 h at 37°C. The levels of PRRSV ORF7 mRNA were detected by qRT-PCR. The data are representative of results from three independent experiments. Error bars indicate the means ± SD (n = 3).
(PDF)

S3 Fig Autophagy benefits PRRSV replication. (**A**) Marc-145 cells were mock or infected with replication-competent (MOI = 0.1) or UV-inactivated (MOI = 0.1) PRRSV for 24 h. The cell lysates were harvested and analyzed by immunoblotting using antibodies against LC3-I/II, PRRSV-N and β-actin. (**B**) Marc-145 cells were transfected with p-mCherry-GFP-LC3 and then infected with live or UV-inactivated PRRSV (MOI = 0.1). The LC3 puncta formation was detected by confocal microscopy. Nuclei were stained with the DNA-binding dye DAPI (blue). Scale bar, 5 μm. (**C**) Quantitation of LC3 puncta formation. Results represent the number of LC3 puncta per cell in panel B (n  =  20). (**D**) Marc-145 cells were mock infected or infected with different doses of PRRSV (MOI = 0.01, 0.1 or 1.0) for 24 h. The cell lysates were harvested and analyzed by immunoblotting using antibodies against LC3-I/II, PRRSV-N and β-actin. (**E**) Marc-145 cells were transfected with p-mCherry-GFP-LC3 and then different doses of PRRSV (MOI = 0.01, 0.1 or 1.0). At 24 hpi, autophagic plaques in the cells was detected by confocal microscopy analysis. Nuclei were stained with the DNA-binding dye DAPI (blue). Scale bar, 5 μm. (**F**) Quantitation of LC3 puncta formation. Results represent the number of LC3 puncta per cell in panel E (n  =  20). (G-H) Marc-145 cell were mock or infected with PRRSV (MOI = 0.1) with rapamycin (100 nM), 3-MA (20 mM) or DMSO treatment for 24 h. (**G**) Cell lysates were collected and determined by immunoblotting with antibodies against LC3-I/II, PRRSV-N and β-actin. (**H**) TCID_50_ of PRRSV in cell supernatants. The protein levels were quantified by Image J and normalized to β-actin. The data are representative of results from three independent experiments. Error bars indicate the mean (± SD), n = 3 in H or n = 20 in C and F. *, p < 0.05; **, p < 0.01; and ***, p < 0.001.
(PDF)

S4 Fig Densitometric quantification of proteins. The protein bands LC3II/I or ATG7 shown in in Fig 3H were quantified using NIH ImageJ software. The data are presented as the relative amount of LC3II/I or ATG7 normalized to the total level of β-actin in each sample from three independent experiments. Error bars indicate the mean (± SD), n = 3. *, p < 0.05; **, p < 0.01; and ***, p < 0.001.
(PDF)

S5 Fig Densitometric quantification of proteins. (A-E) The protein bands in Fig 4A, 4D, 4F, 4I and 4L were separately quantified using NIH ImageJ software shown in A, B, C, D and E. The data are presented as the relative amount of indicated proteins normalized to the total level of β-actin in each sample from three independent experiments. Error bars indicate the mean (± SD), n = 3. *, p < 0.05; **, p < 0.01; and ***, p < 0.001.
(PDF)

S6 Fig The effect of CaMKII on PRRSV infection. (A and B) Marc-145 cells were transfected with siRNA targeting to CaMKII or siNC for 24 h, and mock or infected with PRRRV (MOI = 0.1) for another 24 h. (**A**) Cell lysates were prepared and analyzed by immunoblotting using anti-CaMKII, anti-p-AMPK, anti-AMPK, anti-p-mTOR, anti-mTOR, anti-LC3-I/II, anti PRRSV-N, and anti-β-actin antibodies. (**B**) TCID_50_ of PRRSV in cell supernatants. The data are representative of results from three independent experiments. Error bars indicate the mean (± SD) of three independent experiments. *, p < 0.05; **, p < 0.01; and ***, p < 0.001.
(PDF)

S7 Fig Densitometric quantification of proteins. The protein bands shown in in Fig 5A were quantified using NIH ImageJ software. The data are presented as the relative amount of proteins normalized to the total level of β-actin in each sample from three independent experiments. Error bars indicate the mean (± SD), n = 3. *, p < 0.05; **, p < 0.01; and ***, p < 0.001.
(PDF)

S8 Fig PRRSV induces endoplasmic reticulum calcium depletion in the early stage of infection. (A-B) Kinetics of cytoplasmic and endoplasmic reticulum Ca^2+^ induction. Marc-145 cells were infected with PRRSV at an MOI of 0.1 for 2, 4, 6, 8, 10, 12 h, relative Ca^2+^ in cytoplasm (**A**) and endoplasmic reticulum (**B**) were determined by fluorescence of Fluo-8. Data are expressed as means ± SD (n = 3). *p<0.05; **p < 0.01; ***p < 0.001. The experimental data are representative of results from three independent experiments.
(PDF)

S9 Fig Densitometric quantification of proteins. (A-D) The protein bands in Fig 6A, 6E, 6J and 6Q were separately quantified using NIH ImageJ software shown in A, B, C and D. The data are presented as the relative amount of indicated proteins normalized to the total level of β-actin in each sample from three independent experiments. Error bars indicate the mean (± SD), n = 3. *, p < 0.05; **, p < 0.01; and ***, p < 0.001.
(PDF)

S10 Fig Orai1 and STIM1 are essential for PRRSV-induced Ca^2+^ influx, activation of CaMKII-AMPK-mTOR-LC3II signaling, and PRRSV efficient replication. (A) Western blotting was used to quantitate the level of Orai1 in siOrai1 or siNC-transfected Marc-145 cells. (B) The cell viability of Marc-145 cells transfected with siOrai1 or siNC. (C-F) Marc-145 cells were transfected with siOrai1 or siNC for 24 h, and mock or infected with PRRRV (MOI = 0.1) for another 24 h. (C) Cell lysates were prepared and analyzed by immunoblotting using anti-STIM1, anti-Orai1, anti-GRP78, anti-CaMKII, anti-p-AMPK, anti-AMPK, anti-p-mTOR, anti-mTOR, anti-LC3-I/II, anti PRRSV-N, and anti-β-actin antibodies. The protein bands in panel C were quantified using NIH ImageJ software. The graph data are presented as the relative amount of indicated proteins normalized to the total level of β-actin in each sample from three independent experiments. (D) TCID_50_ of PRRSV in cell supernatants. (E) Cytoplasmic Ca^2+^ and (F) ER Ca^2+^ were determined. (G-J) Marc-145 cells were transfected with siSTIM1 or siNC for 24 h, and mock or infected with PRRRV (MOI = 0.1) for another 24 h. (G) Cell lysates were prepared and analyzed by immunoblotting using anti-STIM1, anti-Orai1, anti-GRP78, anti-CaMKII, anti-p-AMPK, anti-AMPK, anti-p-mTOR, anti-mTOR, anti-LC3-I/II, anti PRRSV-N and anti-β-actin antibodies. The protein bands in panel C were quantified using NIH ImageJ software. The graph data are presented as the relative amount of indicated proteins normalized to the total level of β-actin in each sample from three independent experiments. (H) TCID_50_ of PRRSV in cell supernatants. (I) Cytoplasmic Ca^2+^ and (J) ER Ca^2+^ were determined. All data are representative of results from three independent experiments. Error bars indicate the mean (± SD) of three repeats. *, p < 0.05; **, p < 0.01; and ***, p < 0.001.
(PDF)

S11 Fig The effect of Tetracaine HCL or Porcaine on PRRSV replication. (A and C) Marc-145 cell were mock or infected with PRRSV (MOI = 0.1) with Tetracaine HCL (**A**), Porcaine (**C**) or DMSO treatment for 24 h. Cell lysates were collected and determined by immunoblotting with antibodies against PRRSV-N and β-actin. (B and D) The effect of Tetracaine HCL or Porcaine on Marc-145 cell viability. Marc-145 cells were treated with Tetracaine HCL (**B**) or Porcaine (**D**) at indicated concentrations or DMSO for 24 h. Cells were then analyzed with CCK-8 system. (E-F) Densitometric quantification of proteins. The protein bands in Fig 7A and 7F were separately quantified using NIH ImageJ software shown in E and F. The data are presented as the relative amount of indicated proteins normalized to the total level of β-actin in each sample from three independent experiments. Error bars indicate the mean (± SD), n = 3. *, p < 0.05; **, p < 0.01; and ***, p < 0.001. The data are representative of results from three independent experiments. Error bars indicate the mean (± SD) of three repeats.
(PDF)

S12 Fig PRRSV Nsp2 induces autophagy by endoplasmic reticulum stress at the early stage. (**A**) PRRSV Nsp2 is expressed at the early stage of viral infection. Marc-145 cells were mock or infected with PRRSV-GFP (MOI of 0.1) for 2, 4, 6, 8, 10, 12 h. Fluorescence formation was determined by confocal microscopy. Nuclei were stained with the DNA-binding dye DAPI (blue). Scale bar, 80 μm. (**B**) Marc-145 cells were either uninfected or infected with PRRSV-GFP (MOI of 0.1) for the indicated time points. Cells were harvested and lysed for immunoblotting by using indicated antibodies. The level of protein was quantified using ImageJ 1.8.0 software. The graph data are presented as the relative amount of indicated proteins normalized to the total level of β-actin in each sample are averages from three independent experiments. The significance is indicated by *P < 0.05; **P < 0.01; ***P < 0.001.
(PDF)

S13 Fig Densitometric quantification of proteins. (A-B) The protein bands in Fig 9A and 9D were separately quantified using NIH ImageJ software shown in A and B. The data are presented as the relative amount of indicated proteins normalized to the total level of β-actin in each sample from three independent experiments. Error bars indicate the mean (± SD), n = 3. *, p < 0.05; **, p < 0.01; and ***, p < 0.001.
(PDF)

S14 Fig Ca^2+^ promotes PRRSV replication in pulmonary alveolar macrophages (PAMs). PAMs were infected with different PRRSV strains at an MOI of 0.1 for 24 h. (**A**) Cell lysates were analyzed by Western blotting using antibodies against STIM1, Orai1, GRP78, CaMKII, p-AMPK, AMPK, p-mTOR, mTOR, LC3-I/II, PRRSV-N, and β-actin. The levels of proteins were quantified using ImageJ and normalized to β-actin. The graph data are presented from three independent experiments. **(B**) Cytoplasmic and (**C**) ER Ca^2+^ levels were determined by fluorescence of Fluo-8. The data are representative of results from three independent experiments. Error bars indicate the mean (± SD) of three repeats. *, p < 0.05; **, p < 0.01; and ***, p < 0.001.
(PDF)

## References

[1] Cavanagh D. Nidovirales: a new order comprising Coronaviridae and Arteriviridae. Archives of virology. 1997;142(3):629–33. Epub 1997/01/01. . 9349308

[2] Nelsen CJ, Murtaugh MP, Faaberg KS. Porcine reproductive and respiratory syndrome virus comparison: divergent evolution on two continents. Journal of virology. 1999;73(1):270–80. Epub 1998/12/16. doi: 10.1128/JVI.73.1.270-280.1999 ; PubMed Central PMCID: PMC103831. 9847330 PMC103831

[3] Fang Y, Treffers EE, Li Y, Tas A, Sun Z, van der Meer Y, et al. Efficient -2 frameshifting by mammalian ribosomes to synthesize an additional arterivirus protein. Proceedings of the National Academy of Sciences of the United States of America. 2012;109(43):E2920–8. Epub 2012/10/09. doi: 10.1073/pnas.1211145109 ; PubMed Central PMCID: PMC3491471 application that relates to some aspects of this work. 23043113 PMC3491471

[4] Snijder EJ, Kikkert M, Fang Y. Arterivirus molecular biology and pathogenesis. The Journal of general virology. 2013;94(Pt 10):2141–63. Epub 2013/08/14. doi: 10.1099/vir.0.056341-0 . 23939974

[5] Han J, Rutherford MS, Faaberg KS. The porcine reproductive and respiratory syndrome virus nsp2 cysteine protease domain possesses both trans- and cis-cleavage activities. Journal of virology. 2009;83(18):9449–63. Epub 2009/07/10. doi: 10.1128/JVI.00834-09 ; PubMed Central PMCID: PMC2738230. 19587037 PMC2738230

[6] Fang Y, Snijder EJ. The PRRSV replicase: exploring the multifunctionality of an intriguing set of nonstructural proteins. Virus research. 2010;154(1–2):61–76. Epub 2010/08/11. doi: 10.1016/j.virusres.2010.07.030 ; PubMed Central PMCID: PMC7114499. 20696193 PMC7114499

[7] Ziebuhr J, Snijder EJ, Gorbalenya AE. Virus-encoded proteinases and proteolytic processing in the Nidovirales. The Journal of general virology. 2000;81(Pt 4):853–79. Epub 2000/03/22. doi: 10.1099/0022-1317-81-4-853 . 10725411

[8] Lunney JK, Fang Y, Ladinig A, Chen N, Li Y, Rowland B, et al. Porcine Reproductive and Respiratory Syndrome Virus (PRRSV): Pathogenesis and Interaction with the Immune System. Annual review of animal biosciences. 2016;4:129–54. Epub 2015/12/10. doi: 10.1146/annurev-animal-022114-111025 . 26646630

[9] Han J, Zhou L, Ge X, Guo X, Yang H. Pathogenesis and control of the Chinese highly pathogenic porcine reproductive and respiratory syndrome virus. Veterinary microbiology. 2017;209:30–47. Epub 2017/03/16. doi: 10.1016/j.vetmic.2017.02.020 . 28292547

[10] Hidalgo C. Calcium Rules. Circulation. 2017;135(15):1379–81. Epub 2017/04/12. doi: 10.1161/CIRCULATIONAHA.117.024244 . 28396374

[11] Patergnani S, Danese A, Bouhamida E, Aguiari G, Previati M, Pinton P, et al. Various Aspects of Calcium Signaling in the Regulation of Apoptosis, Autophagy, Cell Proliferation, and Cancer. International journal of molecular sciences. 2020;21(21). Epub 2020/11/12. doi: 10.3390/ijms21218323 ; PubMed Central PMCID: PMC7664196. 33171939 PMC7664196

[12] Høyer-Hansen M, Bastholm L, Szyniarowski P, Campanella M, Szabadkai G, Farkas T, et al. Control of macroautophagy by calcium, calmodulin-dependent kinase kinase-beta, and Bcl-2. Molecular cell. 2007;25(2):193–205. Epub 2007/01/25. doi: 10.1016/j.molcel.2006.12.009 . 17244528

[13] Bagchi P. Endoplasmic reticulum in viral infection. International review of cell and molecular biology. 2020;350:265–84. Epub 2020/03/07. doi: 10.1016/bs.ircmb.2019.10.005 . 32138901

[14] Banerjee A, Czinn SJ, Reiter RJ, Blanchard TG. Crosstalk between endoplasmic reticulum stress and anti-viral activities: A novel therapeutic target for COVID-19. Life sciences. 2020;255:117842. Epub 2020/05/27. doi: 10.1016/j.lfs.2020.117842 ; PubMed Central PMCID: PMC7245231. 32454157 PMC7245231

[15] Qi Z, Chen L. Endoplasmic Reticulum Stress and Autophagy. Advances in experimental medicine and biology. 2019;1206:167–77. Epub 2019/11/30. doi: 10.1007/978-981-15-0602-4_8 . 31776985

[16] Song S, Tan J, Miao Y, Zhang Q. Crosstalk of ER stress-mediated autophagy and ER-phagy: Involvement of UPR and the core autophagy machinery. Journal of cellular physiology. 2018;233(5):3867–74. Epub 2017/08/05. doi: 10.1002/jcp.26137 . 28777470

[17] Kania E, Pająk B, Orzechowski A. Calcium homeostasis and ER stress in control of autophagy in cancer cells. BioMed research international. 2015;2015:352794. Epub 2015/03/31. doi: 10.1155/2015/352794 ; PubMed Central PMCID: PMC4363509. 25821797 PMC4363509

[18] Deretic V, Levine B. Autophagy, immunity, and microbial adaptations. Cell host & microbe. 2009;5(6):527–49. Epub 2009/06/17. doi: 10.1016/j.chom.2009.05.016 ; PubMed Central PMCID: PMC2720763. 19527881 PMC2720763

[19] Lennemann NJ, Coyne CB. Catch me if you can: the link between autophagy and viruses. PLoS pathogens. 2015;11(3):e1004685. Epub 2015/03/27. doi: 10.1371/journal.ppat.1004685 ; PubMed Central PMCID: PMC4374752. 25811485 PMC4374752

[20] Liu Q, Qin Y, Zhou L, Kou Q, Guo X, Ge X, et al. Autophagy sustains the replication of porcine reproductive and respiratory virus in host cells. Virology. 2012;429(2):136–47. Epub 2012/05/09. doi: 10.1016/j.virol.2012.03.022 ; PubMed Central PMCID: PMC7111961. 22564420 PMC7111961

[21] Sun MX, Huang L, Wang R, Yu YL, Li C, Li PP, et al. Porcine reproductive and respiratory syndrome virus induces autophagy to promote virus replication. Autophagy. 2012;8(10):1434–47. Epub 2012/06/29. doi: 10.4161/auto.21159 . 22739997

[22] Olivier M. Modulation of host cell intracellular Ca2+. Parasitology today (Personal ed). 1996;12(4):145–50. Epub 1996/04/01. doi: 10.1016/0169-4758(96)10006-5 . 15275223

[23] Chen X, Cao R, Zhong W. Host Calcium Channels and Pumps in Viral Infections. Cells. 2019;9(1). Epub 2020/01/08. doi: 10.3390/cells9010094 ; PubMed Central PMCID: PMC7016755. 31905994 PMC7016755

[24] Komatsu M, Waguri S, Ueno T, Iwata J, Murata S, Tanida I, et al. Impairment of starvation-induced and constitutive autophagy in Atg7-deficient mice. The Journal of cell biology. 2005;169(3):425–34. Epub 2005/05/04. doi: 10.1083/jcb.200412022 ; PubMed Central PMCID: PMC2171928. 15866887 PMC2171928

[25] Berridge MJ, Bootman MD, Lipp P. Calcium—a life and death signal. Nature. 1998;395(6703):645–8. Epub 1998/10/28. doi: 10.1038/27094 . 9790183

[26] Bai D, Fang L, Xia S, Ke W, Wang J, Wu X, et al. Porcine deltacoronavirus (PDCoV) modulates calcium influx to favor viral replication. Virology. 2020;539:38–48. Epub 2019/11/02. doi: 10.1016/j.virol.2019.10.011 ; PubMed Central PMCID: PMC7112098. 31670218 PMC7112098

[27] Li H, Zhang LK, Li SF, Zhang SF, Wan WW, Zhang YL, et al. Calcium channel blockers reduce severe fever with thrombocytopenia syndrome virus (SFTSV) related fatality. Cell Res. 2019;29(9):739–53. Epub 2019/08/25. doi: 10.1038/s41422-019-0214-z ; PubMed Central PMCID: PMC6796935. 31444469 PMC6796935

[28] Hogan PG, Lewis RS, Rao A. Molecular basis of calcium signaling in lymphocytes: STIM and ORAI. Annual review of immunology. 2010;28:491–533. Epub 2010/03/24. doi: 10.1146/annurev.immunol.021908.132550 ; PubMed Central PMCID: PMC2861828. 20307213 PMC2861828

[29] Kasuya G, Hiraizumi M, Maturana AD, Kumazaki K, Fujiwara Y, Liu K, et al. Crystal structures of the TRIC trimeric intracellular cation channel orthologues. Cell research. 2016;26(12):1288–301. Epub 2016/12/03. doi: 10.1038/cr.2016.140 ; PubMed Central PMCID: PMC5143425. 27909292 PMC5143425

[30] Decuypere JP, Bultynck G, Parys JB. A dual role for Ca(2+) in autophagy regulation. Cell calcium. 2011;50(3):242–50. Epub 2011/05/17. doi: 10.1016/j.ceca.2011.04.001 . 21571367

[31] Ghislat G, Knecht E. Ca^2+^-sensor proteins in the autophagic and endocytic traffic. Current protein & peptide science. 2013;14(2):97–110. Epub 2013/01/12. doi: 10.2174/13892037112139990033 ; PubMed Central PMCID: PMC3664516. 23305313 PMC3664516

[32] Wayman GA, Tokumitsu H, Davare MA, Soderling TR. Analysis of CaM-kinase signaling in cells. Cell calcium. 2011;50(1):1–8. Epub 2011/05/03. doi: 10.1016/j.ceca.2011.02.007 ; PubMed Central PMCID: PMC3236032. 21529938 PMC3236032

[33] Racioppi L, Means AR. Calcium/calmodulin-dependent protein kinase kinase 2: roles in signaling and pathophysiology. The Journal of biological chemistry. 2012;287(38):31658–65. Epub 2012/07/11. doi: 10.1074/jbc.R112.356485 ; PubMed Central PMCID: PMC3442500. 22778263 PMC3442500

[34] Gu Y, Qi B, Zhou Y, Jiang X, Zhang X, Li X, et al. Porcine Circovirus Type 2 Activates CaMMKβ to Initiate Autophagy in PK-15 Cells by Increasing Cytosolic Calcium. Viruses. 2016;8(5). Epub 2016/05/24. doi: 10.3390/v8050135 ; PubMed Central PMCID: PMC4885090. 27213427 PMC4885090

[35] Zhou Y, Frey TK, Yang JJ. Viral calciomics: interplays between Ca2+ and virus. Cell calcium. 2009;46(1):1–17. Epub 2009/06/19. doi: 10.1016/j.ceca.2009.05.005 ; PubMed Central PMCID: PMC3449087. 19535138 PMC3449087

[36] Chen Q, Men Y, Wang D, Xu D, Liu S, Xiao S, et al. Porcine reproductive and respiratory syndrome virus infection induces endoplasmic reticulum stress, facilitates virus replication, and contributes to autophagy and apoptosis. Scientific reports. 2020;10(1):13131. Epub 2020/08/06. doi: 10.1038/s41598-020-69959-z ; PubMed Central PMCID: PMC7403369. 32753633 PMC7403369

[37] Xie B, Zhao M, Song D, Wu K, Yi L, Li W, et al. Induction of autophagy and suppression of type I IFN secretion by CSFV. Autophagy. 2021;17(4):925–47. Epub 2020/03/12. doi: 10.1080/15548627.2020.1739445 PubMed Central PMCID: PMC8078712. 32160078 PMC8078712

[38] Nwokonko RM, Cai X, Loktionova NA, Wang Y, Zhou Y, Gill DL. The STIM-Orai Pathway: Conformational Coupling Between STIM and Orai in the Activation of Store-Operated Ca(2+) Entry. Advances in experimental medicine and biology. 2017;993:83–98. Epub 2017/09/14. doi: 10.1007/978-3-319-57732-6_5 ; PubMed Central PMCID: PMC5921863. 28900910 PMC5921863

[39] Venkatachalam K, van Rossum DB, Patterson RL, Ma HT, Gill DL. The cellular and molecular basis of store-operated calcium entry. Nature cell biology. 2002;4(11):E263–72. Epub 2002/11/05. doi: 10.1038/ncb1102-e263 . 12415286

[40] Cao S, Liu J, Ding G, Shao Q, Wang B, Li Y, et al. The tail domain of PRRSV NSP2 plays a key role in aggrephagy by interacting with 14-3-3ε. Veterinary research. 2020;51(1):104. Epub 2020/08/20. doi: 10.1186/s13567-020-00816-7 ; PubMed Central PMCID: PMC7433210. 32811532 PMC7433210

[41] Catanzaro N, Meng XJ. Induction of the unfolded protein response (UPR) suppresses porcine reproductive and respiratory syndrome virus (PRRSV) replication. Virus research. 2020;276:197820. Epub 2019/11/20. doi: 10.1016/j.virusres.2019.197820 . 31743697

[42] van der Meer Y, van Tol H, Locker JK, Snijder EJ. ORF1a-encoded replicase subunits are involved in the membrane association of the arterivirus replication complex. Journal of virology. 1998;72(8):6689–98. Epub 1998/07/11. doi: 10.1128/JVI.72.8.6689-6698.1998 ; PubMed Central PMCID: PMC109868. 9658116 PMC109868

[43] Voorhees RM, Hegde RS. Structure of the Sec61 channel opened by a signal sequence. Science (New York, NY). 2016;351(6268):88–91. Epub 2016/01/02. doi: 10.1126/science.aad4992 ; PubMed Central PMCID: PMC4700591. 26721998 PMC4700591

[44] Geurts MMG, Clausen JD, Arnou B, Montigny C, Lenoir G, Corey RA, et al. The SERCA residue Glu340 mediates interdomain communication that guides Ca(2+) transport. Proceedings of the National Academy of Sciences of the United States of America. 2020;117(49):31114–22. Epub 2020/11/25. doi: 10.1073/pnas.2014896117 ; PubMed Central PMCID: PMC7733806. 33229570 PMC7733806

[45] Medina DL, Di Paola S, Peluso I, Armani A, De Stefani D, Venditti R, et al. Lysosomal calcium signalling regulates autophagy through calcineurin and ​TFEB. Nat Cell Biol. 2015;17(3):288–99. Epub 2015/02/28. doi: 10.1038/ncb3114 ; PubMed Central PMCID: PMC4801004. 25720963 PMC4801004

[46] Lu Q, Bai J, Zhang L, Liu J, Jiang Z, Michal JJ, et al. Two-dimensional liquid chromatography-tandem mass spectrometry coupled with isobaric tags for relative and absolute quantification (iTRAQ) labeling approach revealed first proteome profiles of pulmonary alveolar macrophages infected with porcine reproductive and respiratory syndrome virus. Journal of proteome research. 2012;11(5):2890–903. Epub 2012/04/11. doi: 10.1021/pr201266z . 22486680

[47] Chen X, Zhang Q, Bai J, Zhao Y, Wang X, Wang H, et al. The Nucleocapsid Protein and Nonstructural Protein 10 of Highly Pathogenic Porcine Reproductive and Respiratory Syndrome Virus Enhance CD83 Production via NF-κB and Sp1 Signaling Pathways. Journal of virology. 2017;91(18). Epub 2017/07/01. doi: 10.1128/jvi.00986-17 ; PubMed Central PMCID: PMC5571251. 28659471 PMC5571251

[48] Zhao Y, Song Z, Bai J, Liu X, Nauwynck H, Jiang P. ZAP, a CCCH-Type Zinc Finger Protein, Inhibits Porcine Reproductive and Respiratory Syndrome Virus Replication and Interacts with Viral Nsp9. Journal of virology. 2019;93(10). Epub 2019/03/15. doi: 10.1128/JVI.00001-19 ; PubMed Central PMCID: PMC6498049. 30867303 PMC6498049

[49] Reed LJ, Muench H. A simple method of estimating fifty per cent endpoints. American journal of epidemiology. 1938;27(3):493–7.

[50] Song Z, Song H, Liu D, Yan B, Wang D, Zhang Y, et al. Overexpression of MFN2 alleviates sorafenib-induced cardiomyocyte necroptosis via the MAM-CaMKIIδ pathway in vitro and in vivo. Theranostics. 2022;12(3):1267–85. Epub 2022/02/15. doi: 10.7150/thno.65716 ; PubMed Central PMCID: PMC8771548. 35154486 PMC8771548

[51] Tian P, Estes MK, Hu Y, Ball JM, Zeng CQ, Schilling WP. The rotavirus nonstructural glycoprotein NSP4 mobilizes Ca2+ from the endoplasmic reticulum. Journal of virology. 1995;69(9):5763–72. Epub 1995/09/01. doi: 10.1128/JVI.69.9.5763-5772.1995 ; PubMed Central PMCID: PMC189437. 7637021 PMC189437

[52] Yan N, Wang Y, Chen Z, Liu A, Li Y, Yang B, et al. Stromal Interaction Molecule 1 Promotes the Replication of vvIBDV by Mobilizing Ca(2+) in the ER. Viruses. 2022;14(7). Epub 2022/07/28. doi: 10.3390/v14071524 ; PubMed Central PMCID: PMC9320076. 35891504 PMC9320076

[53] Treiman M, Caspersen C, Christensen SB. A tool coming of age: thapsigargin as an inhibitor of sarco-endoplasmic reticulum Ca(2+)-ATPases. Trends Pharmacol Sci. 1998;19(4):131–5. Epub 1998/06/05. doi: 10.1016/s0165-6147(98)01184-5 . 9612087

[54] Liu X, Song Z, Bai J, Nauwynck H, Zhao Y, Jiang P. Xanthohumol inhibits PRRSV proliferation and alleviates oxidative stress induced by PRRSV via the Nrf2-HMOX1 axis. Veterinary research. 2019;50(1):61. Epub 2019/09/12. doi: 10.1186/s13567-019-0679-2 ; PubMed Central PMCID: PMC6737628. 31506103 PMC6737628

[55] Park CY, Hoover PJ, Mullins FM, Bachhawat P, Covington ED, Raunser S, et al. STIM1 clusters and activates CRAC channels via direct binding of a cytosolic domain to Orai1. Cell. 2009;136(5):876–90. Epub 2009/03/03. doi: 10.1016/j.cell.2009.02.014 ; PubMed Central PMCID: PMC2670439. 19249086 PMC2670439

[56] Prakriya M, Lewis RS. Store-Operated Calcium Channels. Physiological reviews. 2015;95(4):1383–436. Epub 2015/09/25. doi: 10.1152/physrev.00020.2014 ; PubMed Central PMCID: PMC4600950. 26400989 PMC4600950

