# Supplementary figures and images for: Porcine reproductive and respiratory syndrome virus infection triggers autophagy via ER stress-induced calcium signaling to facilitate virus replication
Source: PLoS Pathog. 2023 Mar 27;19(3):e1011295. doi: 10.1371/journal.ppat.1011295 (PMC10079224; doi:10.1371/journal.ppat.1011295)

S1 Fig

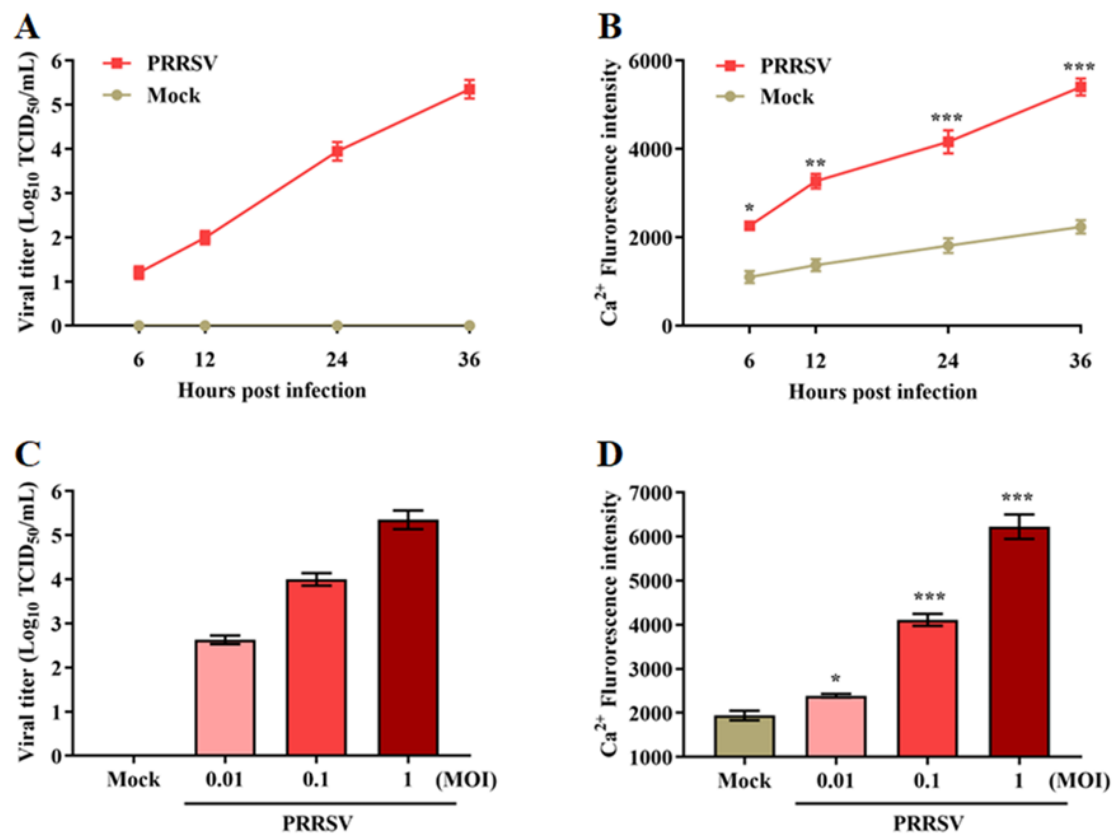

Supplement: S1 Fig — (A-B) Marc-145 cells were infected at MOI of 0.1 for different timepoints (6, 12, 24, 36 h). (A) PRRSV growth kinetics. (B) Increase in cytoplasmic Ca2+ over infection time course. (C-D) Marc-145 cells were infected with PRRSV at different MOIs for 24 h. (C) Viral titers as a function of MOI at 24 hpi. (D) Cytoplasmic Ca2+ as a function of MOI. Data are expressed as means ± SD (n = 3). *p<0.05; **p < 0.01; ***p < 0.001. (PDF) [file ppat.1011295.s001.pdf]

S2 Fig

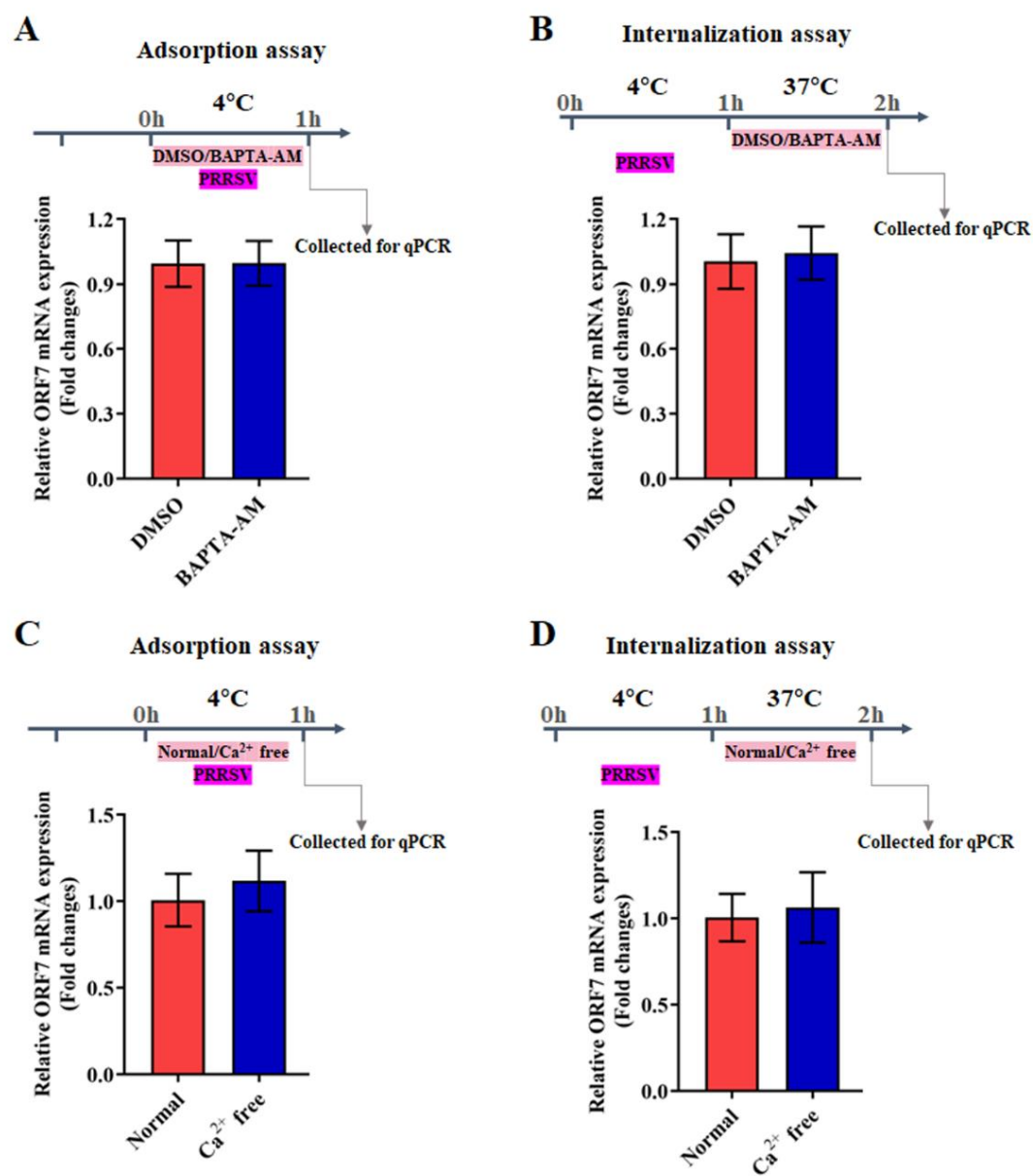

Supplement: S2 Fig — (A-D) The effect of BAPTA-AM or Ca2+ concentration on PRRSV adsorption and internalization. (A and C) Adsorption assay. Cells were incubated with a mixture of BAPTA-AM (50μM)/DMSO (A) or Ca2+ free/Ca2+ medium (C) and PRRSV for 1 h at 4°C and then harvested for qRT-PCR. (B and D) Internalization assay. Cells were incubated with PRRSV (1 MOI) for 1 h at 4°C, washed, and finally incubated with BAPTA-AM (50μM)/DMSO (B) or Ca2+ free/Ca2+ medium (D) for another 1 h at 37°C. The levels of PRRSV ORF7 mRNA were detected by qRT-PCR. The data are representative of results from three independent experiments. Error bars indicate the means ± SD (n = 3). (PDF) [file ppat.1011295.s002.pdf]

S3 Fig

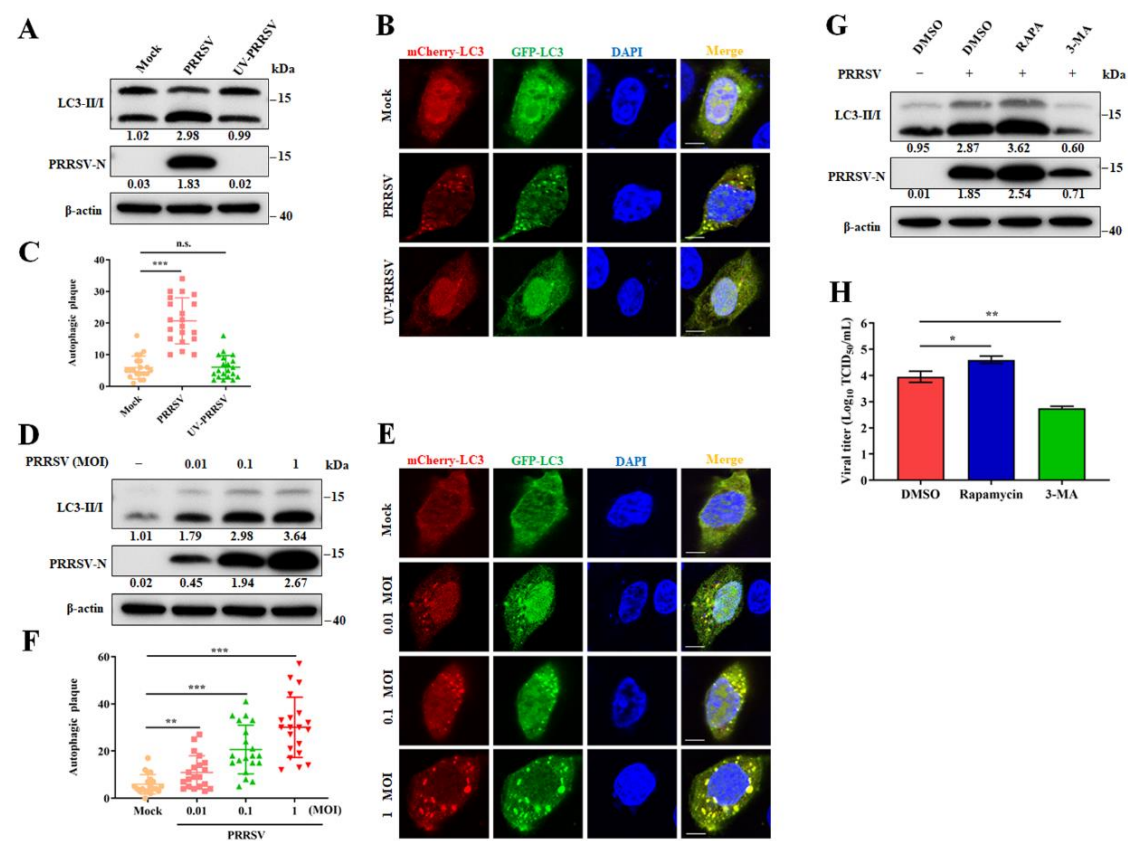

Supplement: S3 Fig — (A) Marc-145 cells were mock or infected with replication-competent (MOI = 0.1) or UV-inactivated (MOI = 0.1) PRRSV for 24 h. The cell lysates were harvested and analyzed by immunoblotting using antibodies against LC3-I/II, PRRSV-N and β-actin. (B) Marc-145 cells were transfected with p-mCherry-GFP-LC3 and then infected with live or UV-inactivated PRRSV (MOI = 0.1). The LC3 puncta formation was detected by confocal microscopy. Nuclei were stained with the DNA-binding dye DAPI (blue). Scale bar, 5 μm. (C) Quantitation of LC3 puncta formation. Results represent the number of LC3 puncta per cell in panel B (n = 20). (D) Marc-145 cells were mock infected or infected with different doses of PRRSV (MOI = 0.01, 0.1 or 1.0) for 24 h. The cell lysates were harvested and analyzed by immunoblotting using antibodies against LC3-I/II, PRRSV-N and β-actin. (E) Marc-145 cells were transfected with p-mCherry-GFP-LC3 and then different doses of PRRSV (MOI = 0.01, 0.1 or 1.0). At 24 hpi, autophagic plaques in the cells was detected by confocal microscopy analysis. Nuclei were stained with the DNA-binding dye DAPI (blue). Scale bar, 5 μm. (F) Quantitation of LC3 puncta formation. Results represent the number of LC3 puncta per cell in panel E (n = 20). (G-H) Marc-145 cell were mock or infected with PRRSV (MOI = 0.1) with rapamycin (100 nM), 3-MA (20 mM) or DMSO treatment for 24 h. (G) Cell lysates were collected and determined by immunoblotting with antibodies against LC3-I/II, PRRSV-N and β-actin. (H) TCID50 of PRRSV in cell supernatants. The protein levels were quantified by Image J and normalized to β-actin. The data are representative of results from three independent experiments. Error bars indicate the mean (± SD), n = 3 in H or n = 20 in C and F. *, p < 0.05; **, p < 0.01; and ***, p < 0.001. (PDF) [file ppat.1011295.s003.pdf]

S4 Fig

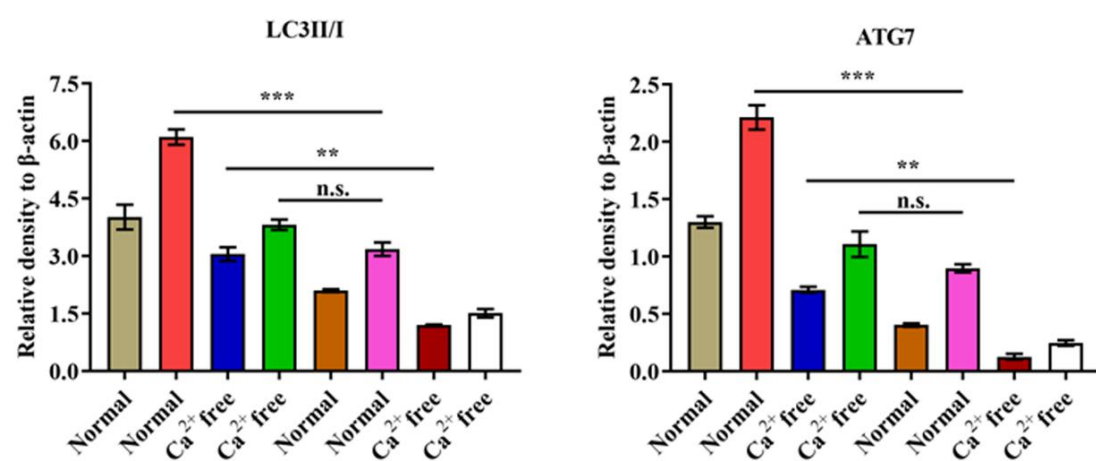

Supplement: S4 Fig — The protein bands LC3II/I or ATG7 shown in in Fig 3H were quantified using NIH ImageJ software. The data are presented as the relative amount of LC3II/I or ATG7 normalized to the total level of β-actin in each sample from three independent experiments. Error bars indicate the mean (± SD), n = 3. *, p < 0.05; **, p < 0.01; and ***, p < 0.001. (PDF) [file ppat.1011295.s004.pdf]

S5 Fig

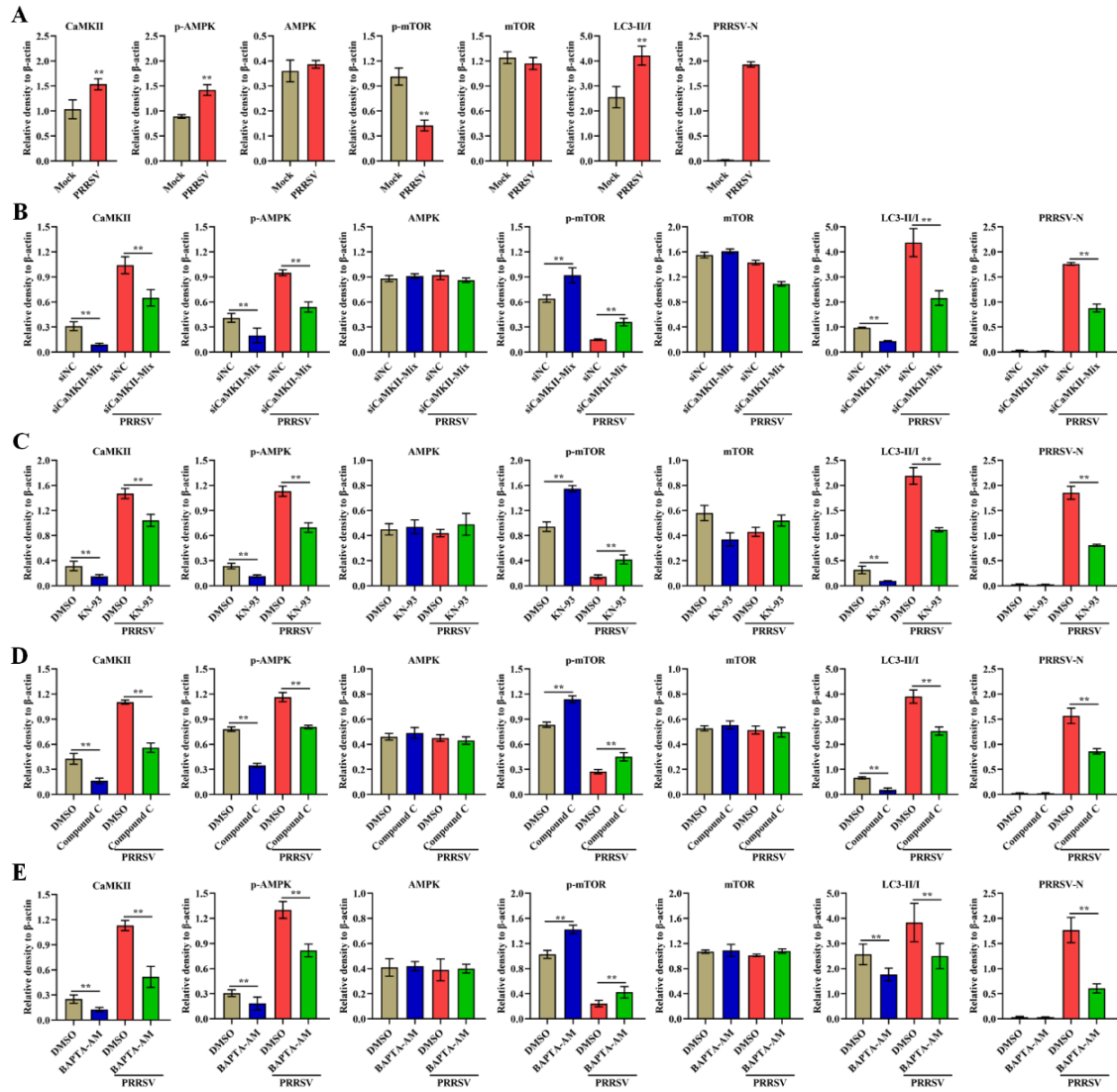

Supplement: S5 Fig — (A-E) The protein bands in Fig 4A, 4D, 4F, 4I and 4L were separately quantified using NIH ImageJ software shown in A, B, C, D and E. The data are presented as the relative amount of indicated proteins normalized to the total level of β-actin in each sample from three independent experiments. Error bars indicate the mean (± SD), n = 3. *, p < 0.05; **, p < 0.01; and ***, p < 0.001. (PDF) [file ppat.1011295.s005.pdf]

S6 Fig

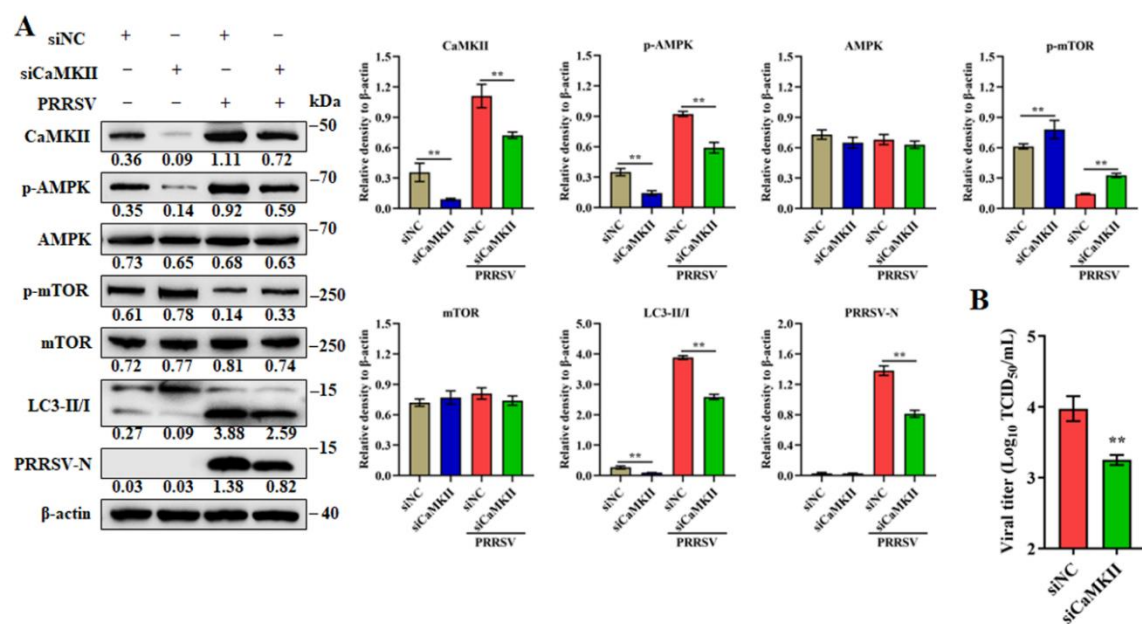

Supplement: S6 Fig — (A and B) Marc-145 cells were transfected with siRNA targeting to CaMKII or siNC for 24 h, and mock or infected with PRRRV (MOI = 0.1) for another 24 h. (A) Cell lysates were prepared and analyzed by immunoblotting using anti-CaMKII, anti-p-AMPK, anti-AMPK, anti-p-mTOR, anti-mTOR, anti-LC3-I/II, anti PRRSV-N, and anti-β-actin antibodies. (B) TCID50 of PRRSV in cell supernatants. The data are representative of results from three independent experiments. Error bars indicate the mean (± SD) of three independent experiments. *, p < 0.05; **, p < 0.01; and ***, p < 0.001. (PDF) [file ppat.1011295.s006.pdf]

S7 Fig

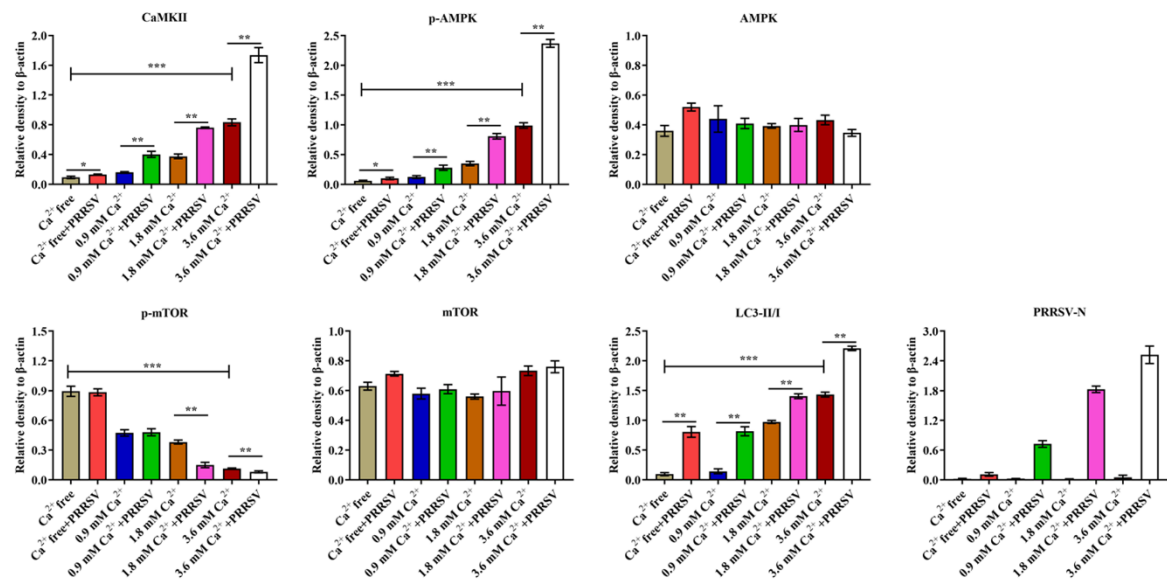

Supplement: S7 Fig — The protein bands shown in in Fig 5A were quantified using NIH ImageJ software. The data are presented as the relative amount of proteins normalized to the total level of β-actin in each sample from three independent experiments. Error bars indicate the mean (± SD), n = 3. *, p < 0.05; **, p < 0.01; and ***, p < 0.001. (PDF) [file ppat.1011295.s007.pdf]

S8 Fig

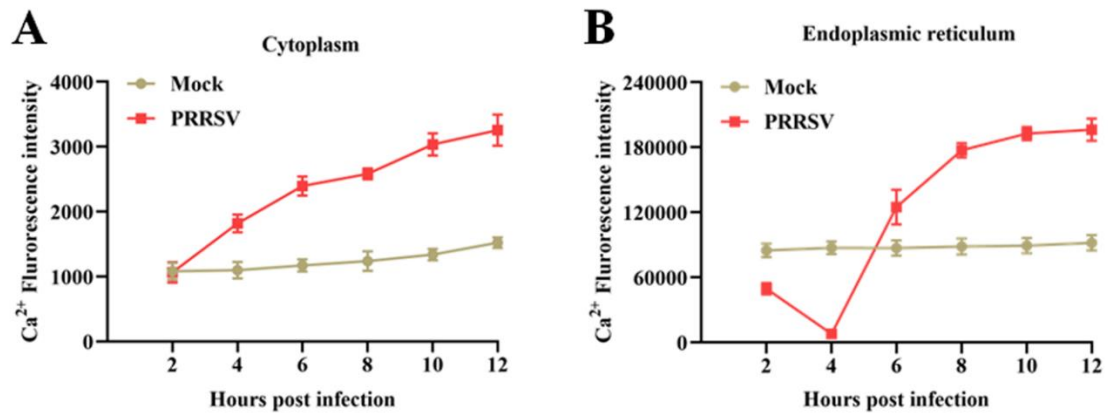

Supplement: S8 Fig — (A-B) Kinetics of cytoplasmic and endoplasmic reticulum Ca2+ induction. Marc-145 cells were infected with PRRSV at an MOI of 0.1 for 2, 4, 6, 8, 10, 12 h, relative Ca2+ in cytoplasm (A) and endoplasmic reticulum (B) were determined by fluorescence of Fluo-8. Data are expressed as means ± SD (n = 3). *p<0.05; **p < 0.01; ***p < 0.001. The experimental data are representative of results from three independent experiments. (PDF) [file ppat.1011295.s008.pdf]

S9 Fig

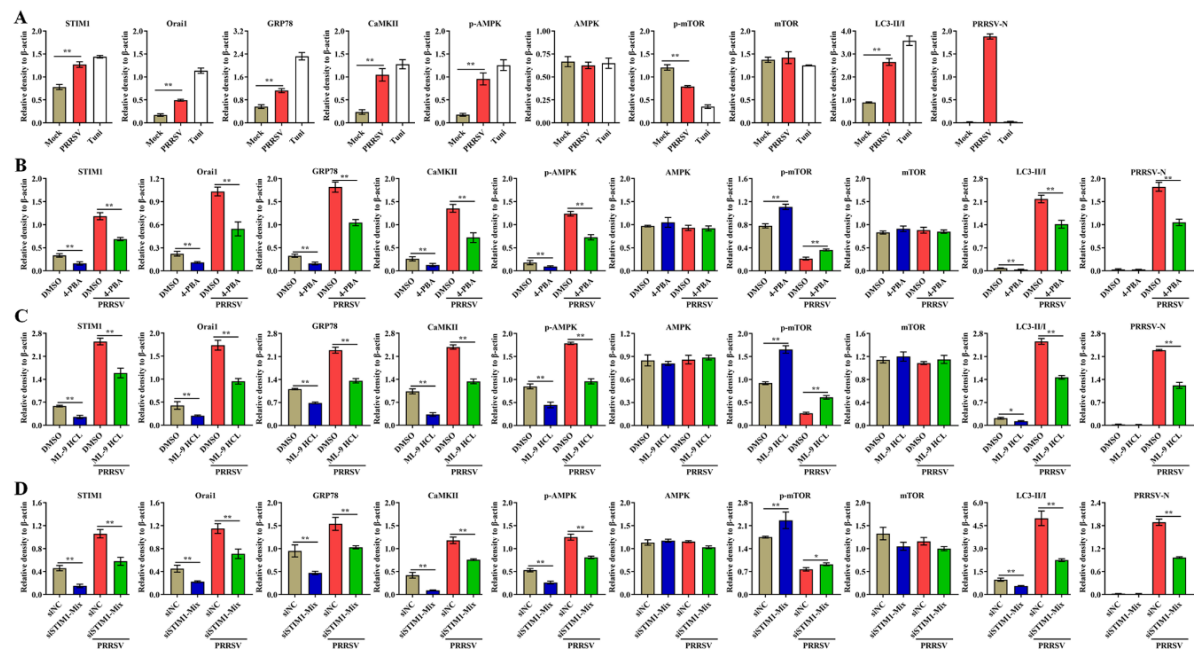

Supplement: S9 Fig — (A-D) The protein bands in Fig 6A, 6E, 6J and 6Q were separately quantified using NIH ImageJ software shown in A, B, C and D. The data are presented as the relative amount of indicated proteins normalized to the total level of β-actin in each sample from three independent experiments. Error bars indicate the mean (± SD), n = 3. *, p < 0.05; **, p < 0.01; and ***, p < 0.001. (PDF) [file ppat.1011295.s009.pdf]

# S10 Fig

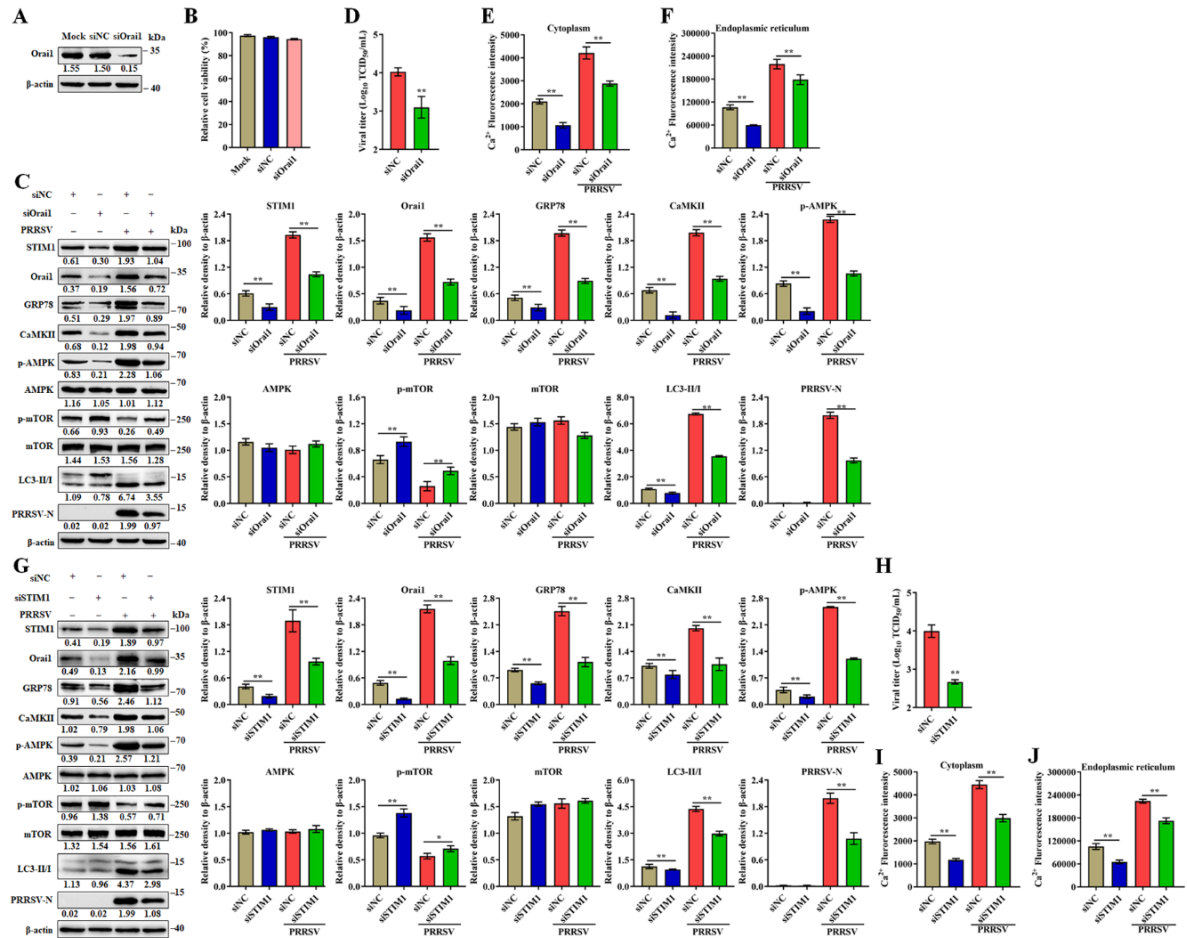

Supplement: S10 Fig — (A) Western blotting was used to quantitate the level of Orai1 in siOrai1 or siNC-transfected Marc-145 cells. (B) The cell viability of Marc-145 cells transfected with siOrai1 or siNC. (C-F) Marc-145 cells were transfected with siOrai1 or siNC for 24 h, and mock or infected with PRRRV (MOI = 0.1) for another 24 h. (C) Cell lysates were prepared and analyzed by immunoblotting using anti-STIM1, anti-Orai1, anti-GRP78, anti-CaMKII, anti-p-AMPK, anti-AMPK, anti-p-mTOR, anti-mTOR, anti-LC3-I/II, anti PRRSV-N, and anti-β-actin antibodies. The protein bands in panel C were quantified using NIH ImageJ software. The graph data are presented as the relative amount of indicated proteins normalized to the total level of β-actin in each sample from three independent experiments. (D) TCID50 of PRRSV in cell supernatants. (E) Cytoplasmic Ca2+ and (F) ER Ca2+ were determined. (G-J) Marc-145 cells were transfected with siSTIM1 or siNC for 24 h, and mock or infected with PRRRV (MOI = 0.1) for another 24 h. (G) Cell lysates were prepared and analyzed by immunoblotting using anti-STIM1, anti-Orai1, anti-GRP78, anti-CaMKII, anti-p-AMPK, anti-AMPK, anti-p-mTOR, anti-mTOR, anti-LC3-I/II, anti PRRSV-N and anti-β-actin antibodies. The protein bands in panel C were quantified using NIH ImageJ software. The graph data are presented as the relative amount of indicated proteins normalized to the total level of β-actin in each sample from three independent experiments. (H) TCID50 of PRRSV in cell supernatants. (I) Cytoplasmic Ca2+ and (J) ER Ca2+ were determined. All data are representative of results from three independent experiments. Error bars indicate the mean (± SD) of three repeats. *, p < 0.05; **, p < 0.01; and ***, p < 0.001. (PDF) [file ppat.1011295.s010.pdf]

S11 Fig

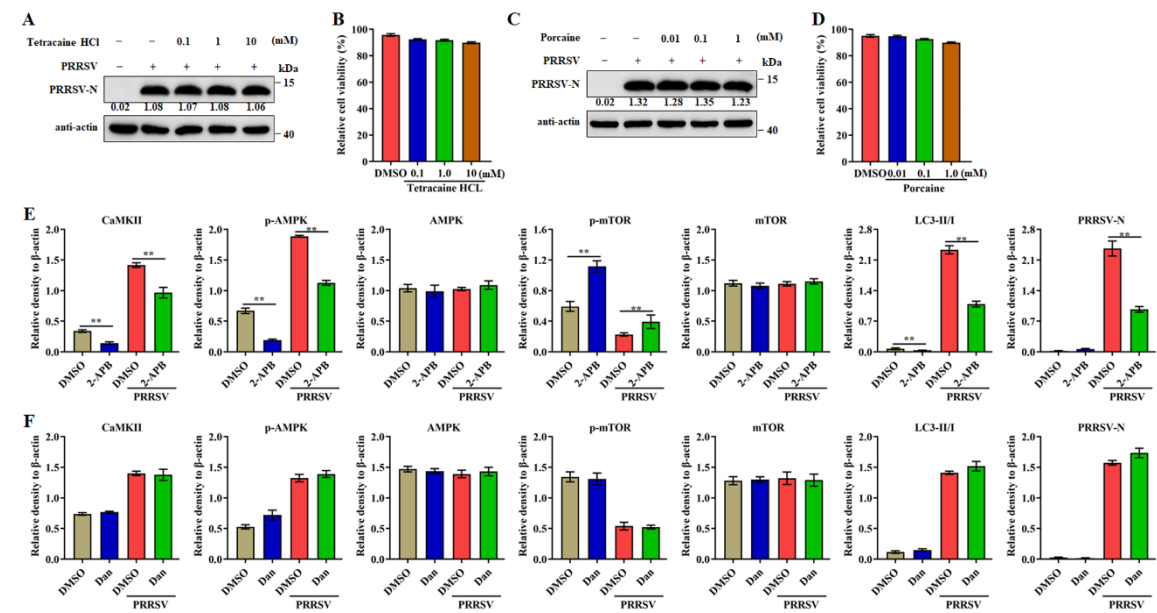

Supplement: S11 Fig — (A and C) Marc-145 cell were mock or infected with PRRSV (MOI = 0.1) with Tetracaine HCL (A), Porcaine (C) or DMSO treatment for 24 h. Cell lysates were collected and determined by immunoblotting with antibodies against PRRSV-N and β-actin. (B and D) The effect of Tetracaine HCL or Porcaine on Marc-145 cell viability. Marc-145 cells were treated with Tetracaine HCL (B) or Porcaine (D) at indicated concentrations or DMSO for 24 h. Cells were then analyzed with CCK-8 system. (E-F) Densitometric quantification of proteins. The protein bands in Fig 7A and 7F were separately quantified using NIH ImageJ software shown in E and F. The data are presented as the relative amount of indicated proteins normalized to the total level of β-actin in each sample from three independent experiments. Error bars indicate the mean (± SD), n = 3. *, p < 0.05; **, p < 0.01; and ***, p < 0.001. The data are representative of results from three independent experiments. Error bars indicate the mean (± SD) of three repeats. (PDF) [file ppat.1011295.s011.pdf]

S12 Fig

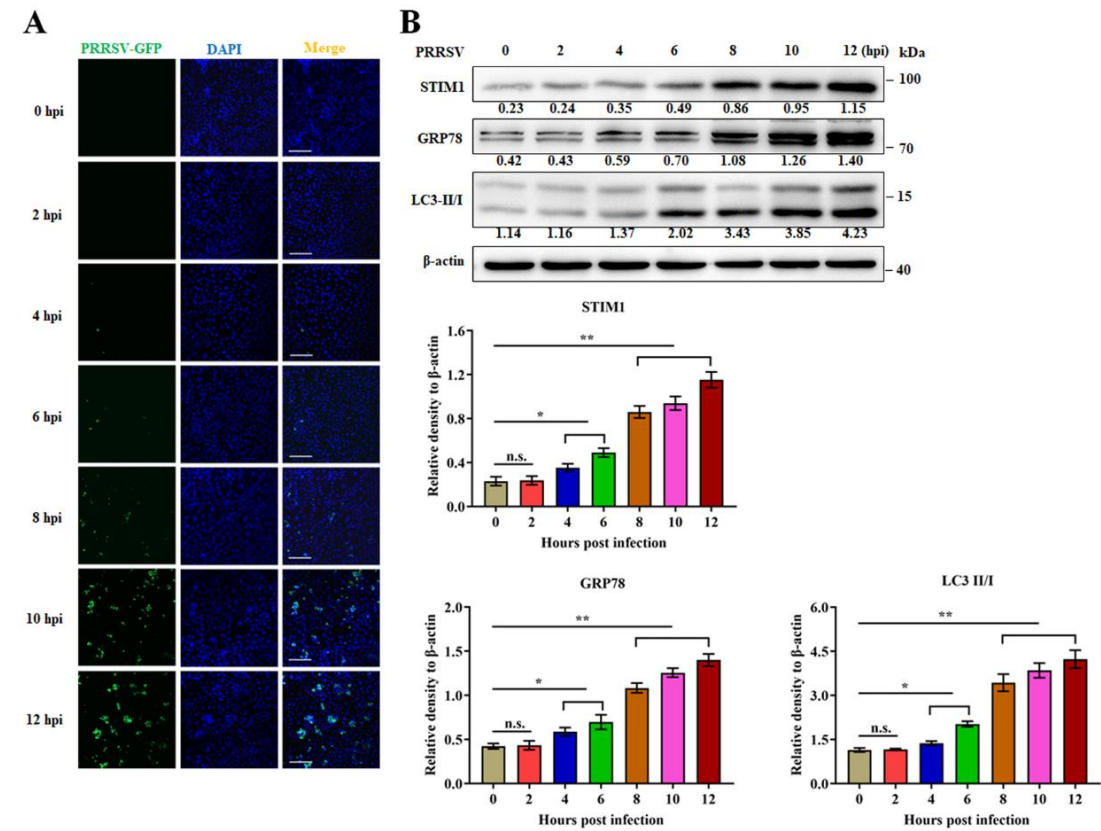

Supplement: S12 Fig — (A) PRRSV Nsp2 is expressed at the early stage of viral infection. Marc-145 cells were mock or infected with PRRSV-GFP (MOI of 0.1) for 2, 4, 6, 8, 10, 12 h. Fluorescence formation was determined by confocal microscopy. Nuclei were stained with the DNA-binding dye DAPI (blue). Scale bar, 80 μm. (B) Marc-145 cells were either uninfected or infected with PRRSV-GFP (MOI of 0.1) for the indicated time points. Cells were harvested and lysed for immunoblotting by using indicated antibodies. The level of protein was quantified using ImageJ 1.8.0 software. The graph data are presented as the relative amount of indicated proteins normalized to the total level of β-actin in each sample are averages from three independent experiments. The significance is indicated by *P < 0.05; **P < 0.01; ***P < 0.001. (PDF) [file ppat.1011295.s012.pdf]

S13 Fig

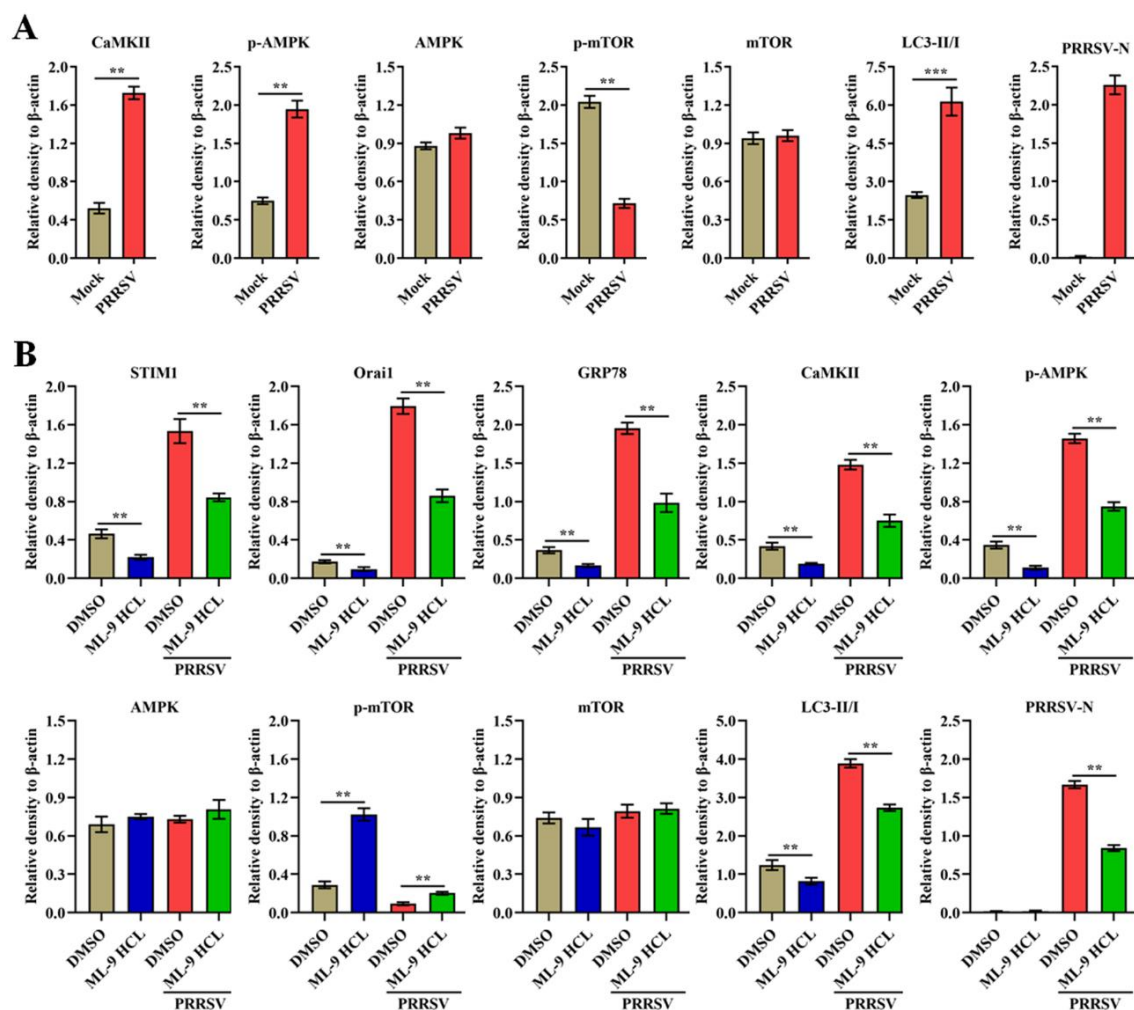

Supplement: S13 Fig — (A-B) The protein bands in Fig 9A and 9D were separately quantified using NIH ImageJ software shown in A and B. The data are presented as the relative amount of indicated proteins normalized to the total level of β-actin in each sample from three independent experiments. Error bars indicate the mean (± SD), n = 3. *, p < 0.05; **, p < 0.01; and ***, p < 0.001. (PDF) [file ppat.1011295.s013.pdf]

S14 Fig

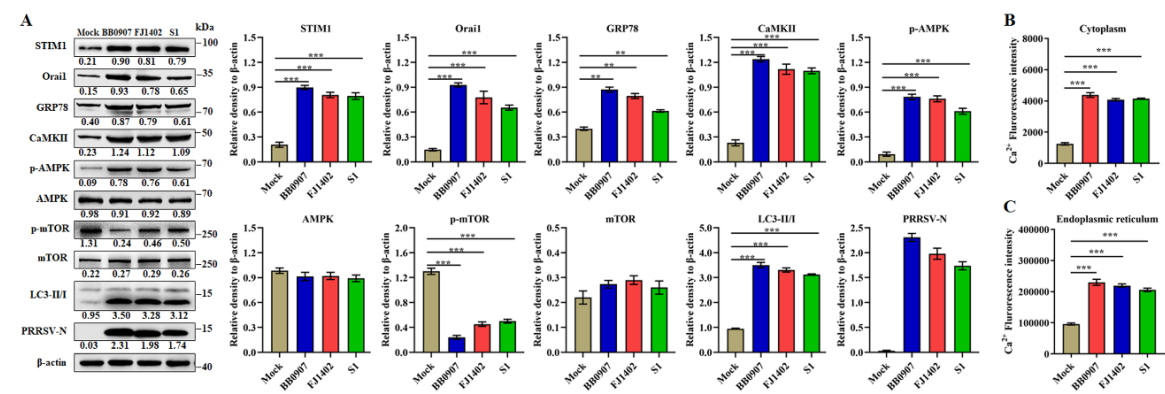

Supplement: S14 Fig — PAMs were infected with different PRRSV strains at an MOI of 0.1 for 24 h. (A) Cell lysates were analyzed by Western blotting using antibodies against STIM1, Orai1, GRP78, CaMKII, p-AMPK, AMPK, p-mTOR, mTOR, LC3-I/II, PRRSV-N, and β-actin. The levels of proteins were quantified using ImageJ and normalized to β-actin. The graph data are presented from three independent experiments. (B) Cytoplasmic and (C) ER Ca2+ levels were determined by fluorescence of Fluo-8. The data are representative of results from three independent experiments. Error bars indicate the mean (± SD) of three repeats. *, p < 0.05; **, p < 0.01; and ***, p < 0.001. (PDF) [file ppat.1011295.s014.pdf]
